# Twists and Turns in the Salicylate Catabolism of *Aspergillus terreus*, Revealing New Roles of the 3-Hydroxyanthranilate Pathway

**DOI:** 10.1128/mSystems.00230-20

**Published:** 2021-01-26

**Authors:** Tiago M. Martins, Celso Martins, Paula Guedes, Cristina Silva Pereira

**Affiliations:** a Instituto de Tecnologia Química e Biológica António Xavier, Universidade Nova de Lisboa (ITQB NOVA), Oeiras, Portugal; b CENSE—Center for Environmental and Sustainability Research, Department of Environmental Sciences and Engineering, NOVA School of Science and Technology, NOVA University Lisbon, Caparica, Portugal; City of Knowledge

**Keywords:** RNA-seq, aromatic compound catabolism, *Aspergillus nidulans*, *Aspergillus terreus*, 3-oxoadipate pathway, gentisate, tryptophan kynurenine pathway, nicotinate catabolism, secondary metabolism, saprophytic *Ascomycota*

## Abstract

In fungi, salicylate catabolism was believed to proceed only through the catechol branch of the 3-oxoadipate pathway, as shown, e.g., in Aspergillus nidulans. However, the observation of a transient accumulation of gentisate upon the cultivation of Aspergillus terreus in salicylate medium questions this concept. To address this, we have run a comparative analysis of the transcriptome of these two species after growth in salicylate using acetate as a control condition. The results revealed the high complexity of the salicylate metabolism in A. terreus with the concomitant positive regulation of several pathways for the catabolism of aromatic compounds. This included the unexpected joint action of two pathways—3-hydroxyanthranilate and nicotinate—possibly crucial for the catabolism of aromatics in this fungus. Importantly, the 3-hydroxyanthranilate catabolic pathway in fungi is described here for the first time, whereas new genes participating in the nicotinate metabolism are also proposed. The transcriptome analysis showed also for the two species an intimate relationship between salicylate catabolism and secondary metabolism. This study emphasizes that the central pathways for the catabolism of aromatic hydrocarbons in fungi hold many mysteries yet to be discovered.

**IMPORTANCE** Aspergilli are versatile cell factories used in industry for the production of organic acids, enzymes, and pharmaceutical drugs. To date, bio-based production of organic acids relies on food substrates. These processes are currently being challenged to switch to renewable nonfood raw materials—a reality that should inspire the use of lignin-derived aromatic monomers. In this context, aspergilli emerge at the forefront of future bio-based approaches due to their industrial relevance and recognized prolific catabolism of aromatic compounds. Notwithstanding considerable advances in the field, there are still important knowledge gaps in the central catabolism of aromatic hydrocarbons in fungi. Here, we disclose a novel central pathway, 3-hydroxyanthranilate, defying previously established ideas on the central metabolism of the aromatic amino acid tryptophan in *Ascomycota*. We also observe that the catabolism of the aromatic salicylate greatly activated the secondary metabolism, furthering the significance of using lignin-derived aromatic hydrocarbons as a distinctive biomass source.

## Abstract (precis)

Aspergilli are versatile cell factories used in industry for the production of organic acids, enzymes, and pharmaceutical drugs. To date, bio-based production of organic acids relies on food substrates.

## INTRODUCTION

In recent years, the production of organic acids through bio-based processes has resurfaced as a sustainable alternative to petroleum-based production. This is the case of the biotechnological production of, e.g., succinic acid, which is already produced at an industrial scale (1). The bio-based production of organic acids is currently shifting from food (corn, beet, cane, etc.) to renewable nonfood feedstocks that may include production by engineered microorganisms (2). Aspergilli are used industrially for the production of organic acids (e.g., citric acid and itaconic acid) and secondary metabolites (e.g., the cholesterol-lowering drug lovastatin), hence seen as ideal candidates for novel production processes (3). Lignin, a heterogeneous aromatic plant polymer, is the second most abundant after cellulose. It constitutes a major underutilized residue from various industrial processes (e.g., >100 million tons per year from Kraft pulping alone) (4). Lignin’s high recalcitrance hinders many biotechnological approaches (5), despite the fact that recent progress in biorefinery can ensure its depolymerization into numerous and structurally diverse aromatic hydrocarbons (5, 6). In nature, lignin depolymerization is mostly accomplished by potent oxidative enzymes secreted by fungi that possess multiple strategies for the catabolism of these aromatic hydrocarbons (7). Fungi can use countless peripheral pathways for their catabolism, all of which converge toward a small number of intermediates that undergo ring cleavage in the central pathways. So far, the better described central pathways are of the intermediates catechol, protocatechuate, hydroxyquinol, homogentisate, and gentisate (8). In the last decade, many of the catabolic genes composing these central pathways have been assigned (8–13). Searching for these genes in the genomes of *Dykaria* revealed that the five central pathways are present in both *Ascomycota* and *Basidiomycota*, sharing simultaneously high similarity but also potentially some speciation (e.g., a distinctive *Basidiomycota* protocatechuate 3,4-dioxygenase) (8). In particular, the existence of specific dioxygenases for gallate, hydroquinone, homoprotocatechuate, and pyrogallol, as described for bacteria, remains unclear to date, despite that these compounds are known to be channeled into the known central pathways (10, 14, 15).

Aspergilli generally display in their genomes the orthologous genes for all the better described central catabolic pathways, with the exception of the gentisate pathway, which is either present, partially present, or absent (8, 16). In addition, the gentisate 1,2-dioxygenase orthologous gene is always organized in a cluster that comprises either all the other pathway genes (designated gentisate cluster) or all except the maleylpyruvate isomerase gene (designated the “gentisate-like cluster”) (8). The presence of the two clusters in several fungal genomes (e.g., Aspergillus niger) is suggestive of different and unknown functional roles. Aspergillus terreus has a gentisate-like gene cluster in its genome, whereas Aspergillus nidulans does not have a gentisate gene cluster or a gentisate 1,2-dioxygenase orthologous gene. In the present study, we undertook a comparative transcriptome analysis of these two species upon their growth in salicylate as the sole carbon and energy source, using acetate as the control condition. The study was complemented by expression analyses of targeted genes and chemical analysis of the potential intermediates.

## RESULTS AND DISCUSSION

**FIG 1:**
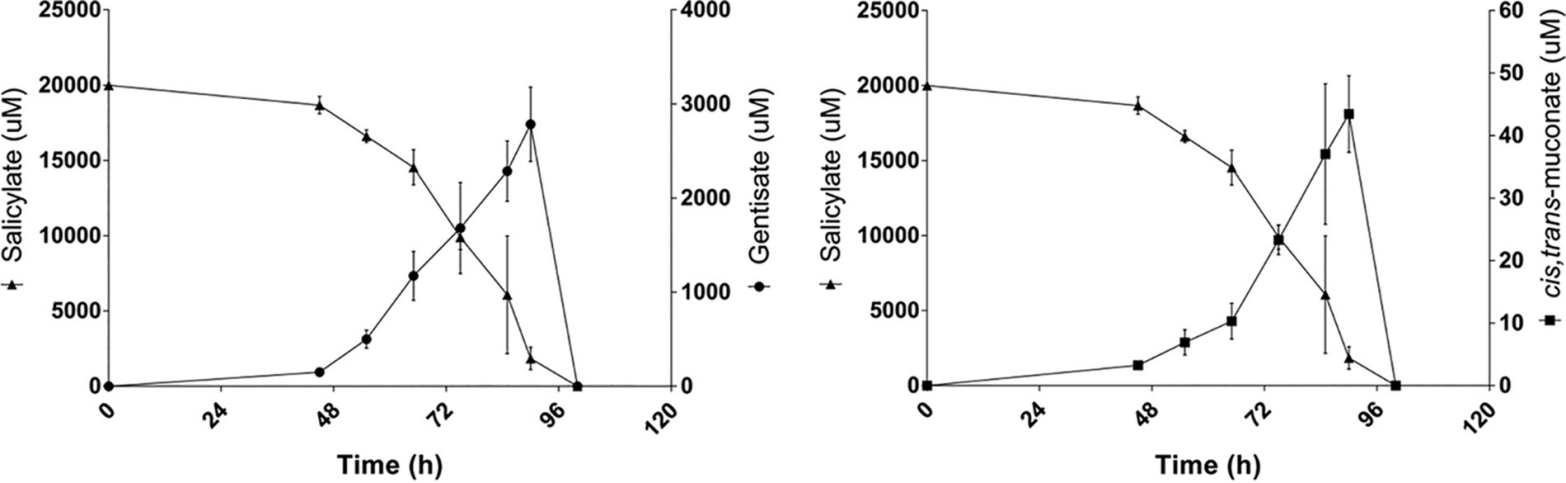
Depletion of salicylate and formation of gentisate and *cis*,*trans*-muconate in Aspergillus terreus liquid medium cultures at discrete cultivation time points (chromatographic analyses). 2,3-Dihydroxybenzoate, of which the retention time is very similar to gentisate, was detected but at very low concentration and therefore not quantified. Values represent the means ± standard deviations from three biological replicates.

**FIG 2:**
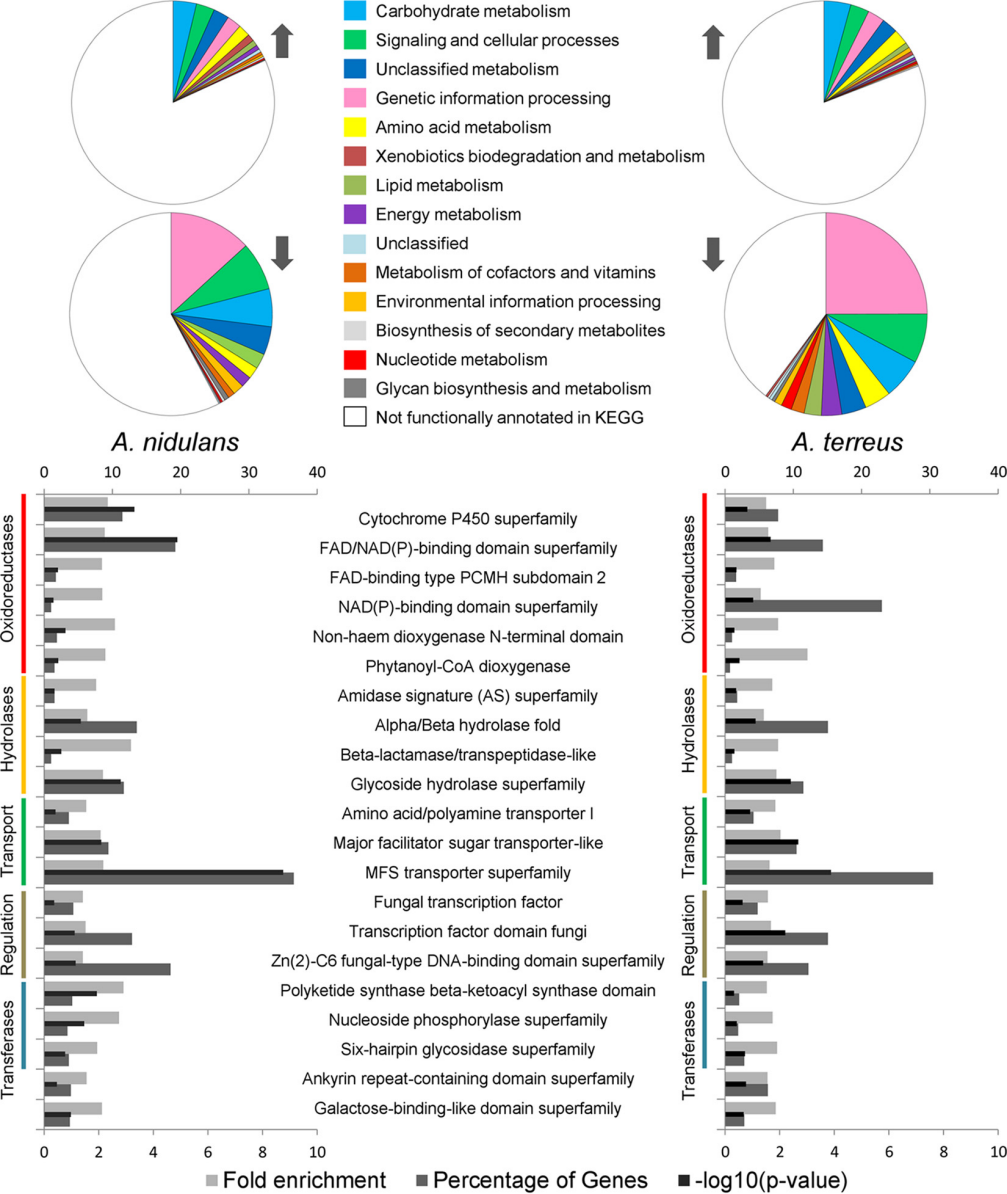
Analysis of the RNA-seq data collected for Aspergillus nidulans and A. terreus grown in salicylate (compared to acetate) comprising functional characterization and protein domains enrichment of the observed differentially expressed genes. KEGG functional categories were slightly simplified by creating super categories that merge the most similar ones. The predicted protein domains of the upregulated and significantly enriched genes in salicylate (*P* ≤ 0.05) for both species are shown. Fold enrichment and percentage of upregulated genes are displayed in the bottom axis and the log_10_ *P* value in the top axis.

Cultivation of Aspergillus terreus in salicylate (20 mM) resulted in the transient accumulation of gentisate up to ca. 3 mM (Fig. 1). This observation conflicts with salicylate catabolism only through the catechol branch of the 3-oxoadipate pathway, as systematically shown in other aspergilli, including in A. nidulans (see reference 9 and references therein). Some yeasts have been shown to use the gentisate pathway for the catabolism of 3-hydroxycinnamates, 3-hydroxybenzoate, and gentisate (2,5-dihydroxybenzoate) (12). The genes of the gentisate pathway are clustered in the genomes of fungi (8), yet in the gentisate-like cluster, the maleylpyruvate isomerase gene is absent, as observed in A. terreus (ATEG_06711 to ATEG_06714). This suggests that during the catabolism of salicylate in A. terreus, gentisate catabolism also takes place through a yet unknown pathway. To better understand the catabolism of salicylate in A. terreus, we analyzed its transcriptome response, as well as that of A. nidulans, grown in salicylate. The comparative analysis revealed that both species share a global common response to salicylate compared to the control conditions in acetate (Fig. 2). In the presence of salicylate, 2,703 encoding genes were found differentially upregulated and 1,489 downregulated in A. nidulans and 2,980 were upregulated and 2,328 downregulated in A. terreus (see Fig. S1 for principal-component analysis [PCA] and MA plots and Tables S4 and S5 in the supplemental material). The first striking feature is that many of the upregulated genes could not be annotated in KEGG (ca. 80%), while half of the downregulated ones could (ca. 50%) (Fig. 2). This is likely related to the fact that well-known processes/pathways were globally downregulated in salicylate medium, such as carbohydrate metabolism (e.g., tricarboxylic acid [TCA] cycle, glycolysis, and pentose phosphate pathways), energy metabolism (e.g., oxidative phosphorylation) and genetic information processing (e.g., protein processing in the endoplasmic reticulum). As expected, the acetate-utilizing genes (*facA* to *facC*, *acuD* to *acuN*, and *maeA* to *maeB*) were downregulated, including the ones from the glyoxylate shunt (Tables S4 and S5). Globally, the amino acid metabolism was greatly affected in both species. Specifically, the aromatic amino acid metabolism was upregulated and their biosynthesis was downregulated (Tables S4 and S5). The peripheral pathways of the catabolism of aromatic compounds show resemblance to the metabolism of phenylalanine, tyrosine, and tryptophan, suggestive of their upregulation. In agreement, and expectably, the xenobiotics’ biodegradation and metabolism were also upregulated. The analysis of the protein domains demonstrated that those upregulated and enriched are from families of oxidoreductases, hydrolases, transporters, transcriptional regulators, and transferases (Fig. 2). On the contrary, none of the downregulated genes encode abundant enriched protein domain families, with the exception of the flavin adenine dinucleotide (FAD)/NAD(P)-binding domain (IPR023753) found in diverse oxidoreductases (e.g., thioredoxin and glutathione reductases and dihydrolipoyl dehydrogenase).

10.1128/mSystems.00230-20.1 FIG S1 Baseline statistical representation of the RNA-seq data collected for Aspergillus nidulans (A) and Aspergillus terreus (B) samples. The principal-component analysis (top), shows the distribution of the whole-transcriptome reads of each of the analyzed replicas (A, acetate medium; S, salicylate medium). In the MA plot (bottom), it is possible to visualize the differences between the measurements taken between the expression of genes in acetate medium and salicylate medium. Both plots were computed using the tools embedded in the DEseq2 package. Download FIG S1, PDF file, 0.3 MB. Copyright © 2021 Martins et al. 2021 Martins et al. This content is distributed under the terms of the Creative Commons Attribution 4.0 International license.

### Secondary metabolism.

Both *Aspergillus* species grown in salicylate underwent similar transcriptome responses in secondary metabolism (SM), with the upregulation of nearly half of the core biosynthesis genes, whereas only ca. 10% underwent downregulation (Table 1; see Table S3 in the supplemental material). Specifically, in A. nidulans, 5 out of 10 *laeA*-like methyltransferase genes (*llmA*, *llmC*, *llmE*, *llmI*, and *llmJ*) with potential regulatory roles in SM (19) were found to be upregulated, while the remaining five were unaltered. In A. terreus, out of the eight *lae*A-like genes, three were upregulated (ATEG_01440, ATEG_06013, and ATEG_09440), four were unaltered (ATEG_00678, ATEG_01936, ATEG_04864, and ATEG_00912), and only one was downregulated (ATEG_07798). Collectively, the concerted response observed in SM reveals its close relation with the catabolism of the aromatic salicylate.

**TABLE 1:** Observed regulation for secondary metabolism biosynthesis genes in salicylate medium

Gene regulation^*a*^	No. of SM biosynthesis genes up- or downregulated in^*b*^:					
	A. nidulans			A. terreus		
	Total	Up	Down	Total	Up	Down
Biosynthesis cluster genes	446	260	39	398	118	51
Core biosynthesis genes	96	57	13	111	41	11
PKS	32	23	3	32	13	3
NRPS	33	17	6	39	13	4
Hybrid	1	1	0	1	1	0
PTases	7	6	0	11	4	1
Terpene	11	6	1	10	1	1
β-Lactone	6	2	2	4	2	1
Cyclic peptide	6	2	1	13	6	1
Siderophore	0	0	0	1	1	0

a PKS, polyketide synthase; NRPS, nonribosomal peptide synthetase; Hybrid, PKS and NRPS; and PTases, aromatic prenyltransferases.

b Genes were considered upregulated (Up) when the fold change was  ≥1, downregulated (Down) with a fold change of ≤−1, and both when the *P* value was ≤0.05.

In salicylate, the monodictyphenone gene cluster of A. nidulans was upregulated, whereas that of sterigmatocystin was downregulated, similar to what was reported before using proteome analyses (9). The monodictyphenone biosynthesis relies on malonyl-coenzyme A (malonyl-CoA) as a precursor (20), formed from plentiful acetyl-CoA made available by the catabolism of salicylate. Besides the monodictyphenone gene cluster, the other known prenyl xanthone biosynthesis genes were also found moderately to highly expressed and upregulated. This included the aromatic DMATS-type prenyltransferase (PTase) genes *xptA* (AN6784) and *xptB* (AN12402), the GMC oxidoreductase superfamily gene *xpt*C (AN7998), and the short-chain dehydrogenase/reductase (SDR) gene AN7999. The last was reported before to have no obvious effect on prenyl xanthone biosynthesis (20). Three DMATS-type PTases were also found moderately to highly expressed and upregulated, including those of the nidulanin A cluster (AN11080) and the viridicatin/aspoquinolone cluster (AN11194/AN11202), which use as precursors either kynurenine or anthranilate, and the amino acids phenylalanine and valine, respectively.

In A. terreus, salicylate greatly upregulated the asterriquinone and the aspulvinone clusters, comprising the DMATS-type PTases ATEG_09980 and ATEG_01730, respectively. These clusters use as precursors indole-3-pyruvate and 4-hydroxyphenylpyruvate, respectively (21–23), which belong to the tryptophan and the phenylalanine pathways also undergoing upregulation in salicylate. A putative cyclic peptide (ribosomally synthesized and posttranslationally modified peptide [RiPP]) gene cluster underwent massive expression and upregulation in salicylate, of which the gene ATEG_07504 encodes the precursor protein.

Collectively, these findings highlight the interconnection of SM and salicylate catabolism in aspergilli, possibly a consequence of shared regulatory and metabolic routes, including the availability of specific precursors. This deserves further consideration in the near future, especially as the use of simple hydrocarbon aromatics, such as salicylate, can help to disclose unknown biosynthesis clusters and the resultant metabolites.

### Salicylate catabolism in Aspergillus nidulans.

As previously reported, salicylate catabolism in A. nidulans most likely results, either directly or via 2,3-dihydroxybenzoate as an intermediate, in the formation of catechol (9), a central substrate for a ring cleavage dioxygenase, which is then channeled to the respective branch of the 3-oxoadipate pathway. This pathway was found mostly upregulated (see Fig. S2 and Table S4 in the supplemental material). However, only the two genes of the non-oxidative decarboxylation pathway and the catechol 1,2-dioxygenase (AN7418, *dhbD*, and AN4532) underwent major expression, while the remaining ones showed only minor expression (AN2114, AN4061, AN4531, and AN10495). We have previously observed that some of these less-expressed genes are not essential for growth in salicylate (9). Accordingly, we previously suggested that the hydroxyquinol variant of the 3-oxoadipate could constitute an alternative path (9). In the present study, we observed by reverse transcription-quantitative PCR (RT-qPCR) expression analyses of the ΔAN4531 mutant grown on salicylate that this alternative path is unlikely (see Fig. S3 in the supplemental material). We also noticed that the dark-colored metabolite (likely 3-oxoadipate enol-lactone that accumulated during the growth of this mutant [9]) disappeared after prolonged growth incubations. These observations suggest that muconate and its catabolic derivatives may be metabolized by broad-substrate-specificity enzymes. The upregulation of several oxidoreductase and hydrolase gene families (Fig. 2) most likely contributes to redundancy in the metabolism of muconate and may offer metabolic plasticity in similar contexts. Besides the two salicylate monooxygenase genes of the catabolic pathway (AN2114 and AN7418), 45 other aromatic-ring FAD-binding monooxygenase genes (IPR002938) were found moderately to highly expressed and upregulated, including seven phenol monooxygenase genes (IPR038220). A major facilitator superfamily (MFS) transporter gene (AN10845) that underwent upregulation in salicylate is located contiguous to *dhbD*. These two genes show shared synteny within aspergilli and penicillia, suggestive that this MFS transporter can be a 2,3-dihydroxybenzoate transporter. This requires validation since numerous other genes of this superfamily were also positively regulated.

10.1128/mSystems.00230-20.2 FIG S2 Diagram of the catabolism of salicylate in A. nidulans. The differentially expressed genes (RNA-seq) regulation and abundance is represented by circles of various sizes and colors (see legend). Download FIG S2, TIF file, 0.5 MB. Copyright © 2021 Martins et al. 2021 Martins et al. This content is distributed under the terms of the Creative Commons Attribution 4.0 International license.

10.1128/mSystems.00230-20.3 FIG S3 Gene expression of metabolic pathways of A. nidulans A1147 wild type (light gray bar) versus ΔAN4531 deletion mutant (dark gray bar) in salicylate (50 mM) compared to the control in acetate (150 mM) (white bar) at 5 days of incubation in solid media. The central intermediates of the respective metabolic pathways are indicated above the corresponding gene’s expression. Genes analyzed: AN7418, salicylate 1-monooxygenase; AN6723, 2,3-dihydroxybenzoate carboxylyase; AN4532, catechol 1,2-dioxygenase; AN4061, muconolactone isomerase; AN3895, muconate cycloisomerase; AN4531, 3-oxoadipic enol-lactone hydrolase; AN0764 and AN9363, hydroxyquinol 1,2-dioxygenase; and AN5178, maleylacetate reductase. Values represent means ± standard error of the means from three biological replicates (*, *P* < 0.05; **, *P* < 0.005). Download FIG S3, TIF file, 0.7 MB. Copyright © 2021 Martins et al. 2021 Martins et al. This content is distributed under the terms of the Creative Commons Attribution 4.0 International license.

One remarkable observation is that we found also a cluster of four genes highly expressed and upregulated in salicylate. It comprises the following genes: the putative salicylate 1-monooxygenase (AN2114) previously identified by us (9), a domain of unknown function (DUF3425) gene (AN2115), a NAD-dependent epimerase/dehydratase (AN2116), and an amidohydrolase (AN2118) (Table S4). The last gene has sequence homology to *orsB* (orsellinic cluster) and *dhbD*, both encoding enzymes that catalyze the decarboxylation of aromatic hydrocarbons. This cluster is only present in the genomes of aspergilli belonging to the *Nidulantes* section (see Fig. S4 in the supplemental material), being absent in most aspergilli, including A. terreus, hence suggestive of a high specific functional role. One hypothesis is that this putative salicylate 1-monooxygenase plays other functions besides the hydroxylation of salicylate in this fungus, possibly acting in other peripheral pathways or in secondary metabolism.

10.1128/mSystems.00230-20.4 FIG S4 The salicylate 1-monooxygenase gene cluster in *Aspergillus* section *Nidulantes*. Here are depicted selected examples of the cluster identified within annotated genomes through sequence homology searches (MultiGeneBlast software). Genes within the cluster are represented using a color code. The differentially expressed genes’ (RNA-seq) regulation and abundance is represented by bars of different scales and colors (upregulated in green, downregulated in red, and RPKM in gray). Download FIG S4, TIF file, 0.3 MB. Copyright © 2021 Martins et al. 2021 Martins et al. This content is distributed under the terms of the Creative Commons Attribution 4.0 International license.

### Salicylate catabolism in Aspergillus terreus.

**FIG 3:**
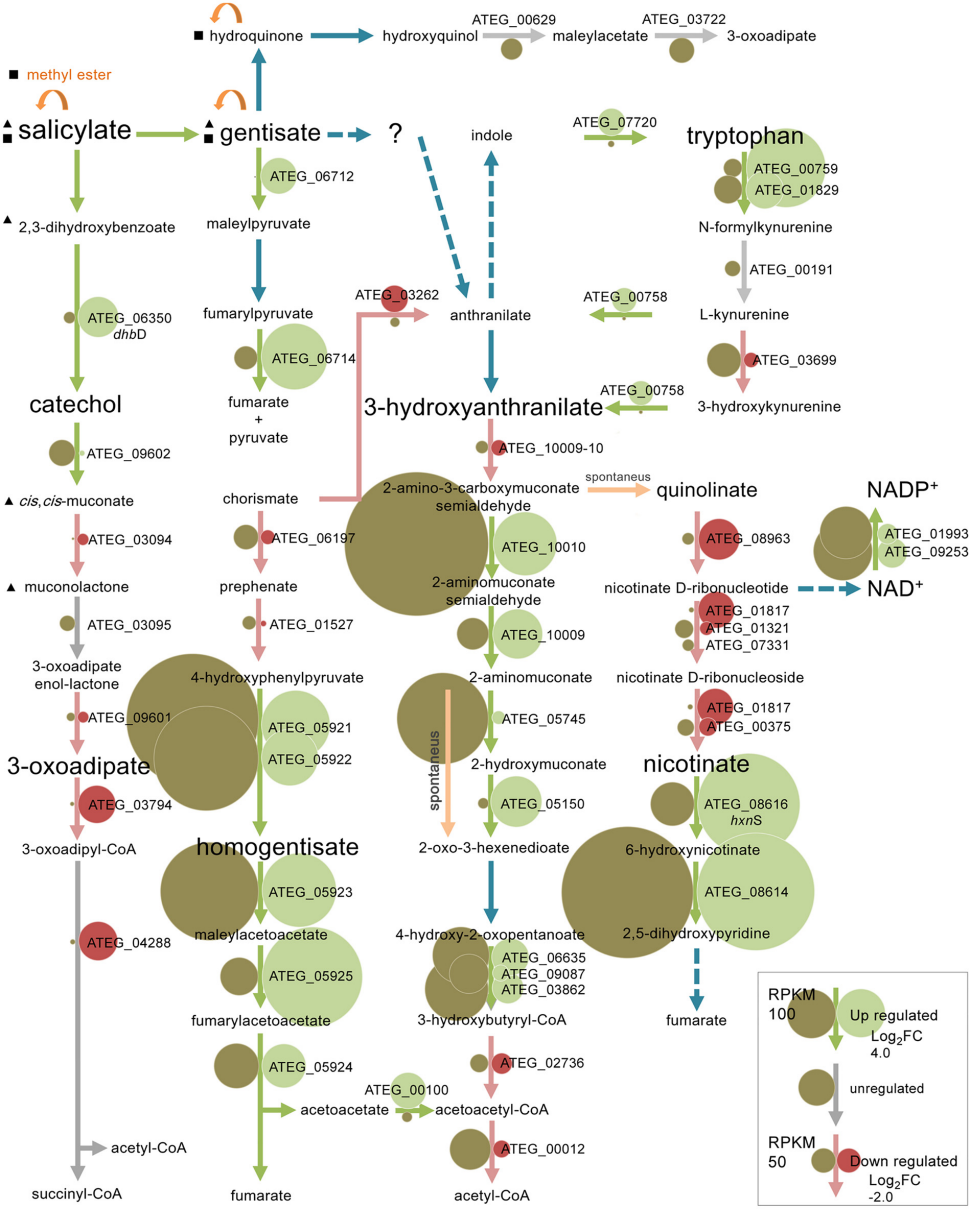
Diagram of the metabolism of salicylate in Aspergillus terreus. The differentially expressed genes’ (RNA-seq) regulation and abundance are represented by circles of various sizes and colors (see the inset legend). Triangles and squares indicate identification by liquid chromatography (LC) and GC-TOF MS, respectively.

**FIG 4:**
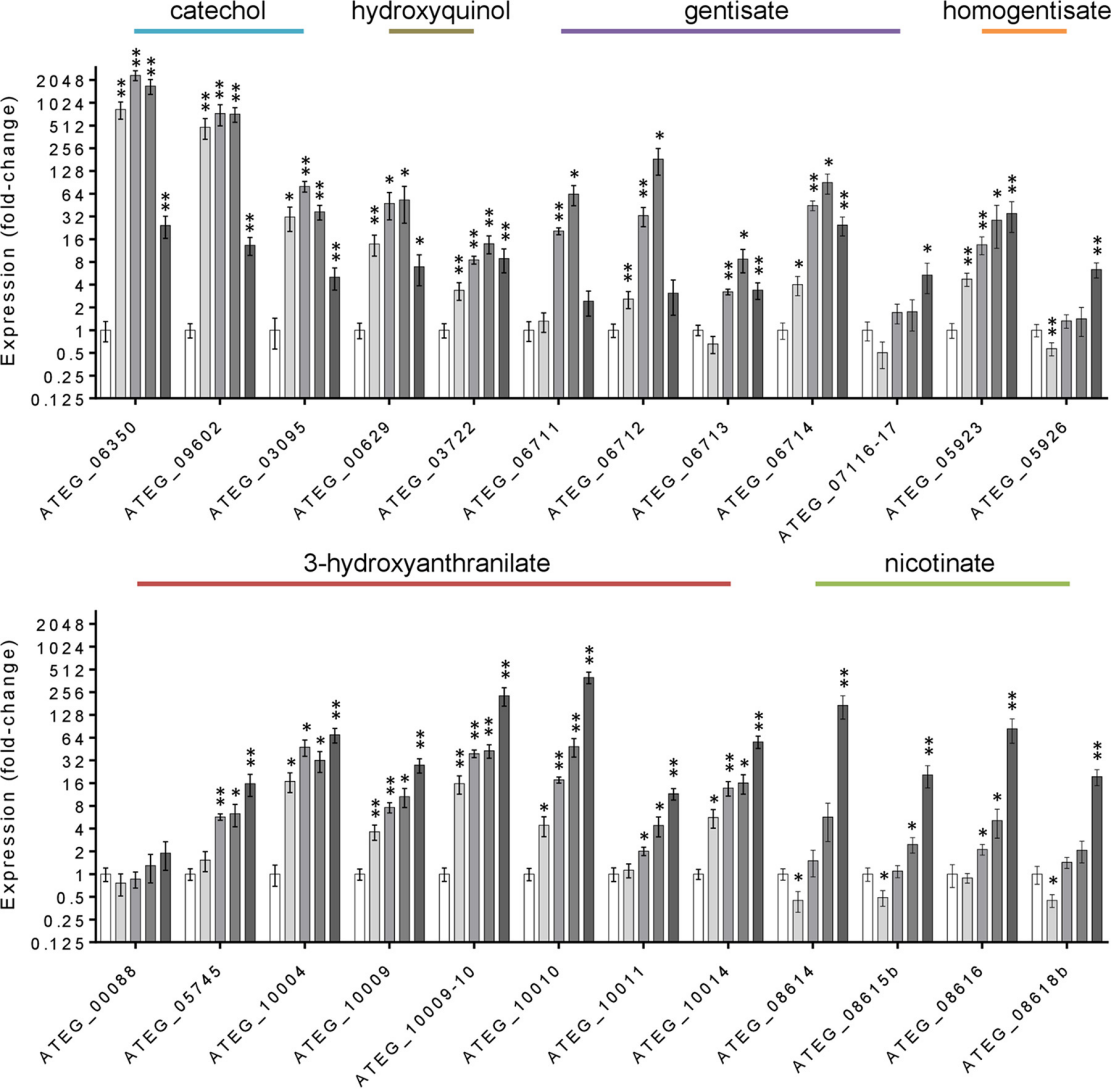
Expression levels of genes coding in the metabolic pathways of Aspergillus terreus in salicylate medium along cultivation (gray bars represent 55, 75, and 90 h and the last time point used for RNA-seq) compared to the control in acetate medium (white bars represent the time point used for RNA-seq). The central intermediates of each metabolic pathway are indicated. Values represent means ± standard errors of the means from three biological replicates: *, *P* < 0.05; and **, *P* < 0.005.

The known pathways in microorganisms for the catabolism of salicylate involve either the catechol branch of the 3-oxoadipate pathway or the gentisate pathway (7). We observed in A. terreus that salicylate was hydroxylated to 2,3-dihydroxybenzoate, similarly to what occurs in A. nidulans, but also to gentisate in significant amounts (Fig. 1). The orthologous or predicted genes for these pathways are present in the genome of A. terreus. The transcriptome sequencing (RNA-seq) data revealed that these genes were mostly unaltered in salicylate medium at the respective time point (Fig. 3; Table S5). However, massive upregulation of the non-oxidative decarboxylase gene (ATEG_06350) and key genes coding in the catechol branch (ATEG_09602 and ATEG_03095) took place at early time points during the incubation in salicylate medium, as verified by their RT-qPCR expression levels (Fig. 4). Corroborating this result, muconolactone, *cis*,*trans*-muconate, and 2,3-dihydroxybenzoate (Fig. 1) were detected in small amounts in the extracellular medium. Small amounts of hydroquinone and methyl ester conjugates of salicylate, gentisate, and hydroquinone were detected as well (see Fig. S5 in the supplemental material). An additional compound was noticed showing characteristics that suggest it constitutes an anthranilate derivative, regardless of the fact that its precise identity remains doubtful (Fig. S5).

10.1128/mSystems.00230-20.5 FIG S5 Chromatographic analysis of Aspergillus terreus ethyl acetate extracts. (A) HPLC chromatograms of the controls (acetate, bottom) and of three replicas of salicylate cultures at 85 h, denoting the compounds showing the highest peak intensity. Their UV-Vis spectra (190 to 400 nm) are shown together with that of pure anthranilic acid: compound 1, *cis,trans*-muconic acid; compound 2, unknown compound with maxima of 218 and 341 nm; compound 3, anthranilic acid standard with maxima of 193, 218, and 333 and retention time similar to gentisic acid; compound 4, gentisic acid; compound 5, 2,3-dihydroxybenzoic acid; compound 6, unknown compound with maxima of 220, 254, and 302 nm; compound 7, unknown compound with maxima of 207 and 317 nm; and compound 8, salicylic acid. (B) GC-TOF MS putative identifications based on mass spectral searches on the National Institute of Standards and Technology and Wiley libraries and on retention index (RI) comparisons. One representative replicate for each of the time points analyzed (65, 75, and 85 h), targeting compounds not present in the control acetate, is shown. Their corresponding GC-TOF MS spectra are shown on the bottom depicting in blue the library compounds’ mass spectra and in red the experimental data. CAS, Chemical Abstracts Service registry number. Download FIG S5, TIF file, 1.1 MB. Copyright © 2021 Martins et al. 2021 Martins et al. This content is distributed under the terms of the Creative Commons Attribution 4.0 International license.

The upregulation of the gentisate-like cluster (ATEG_06711 to ATEG_06714) was less expressive and occurred at later time points compared to the catechol branch (Fig. 4). The salicylate 3-monooxygenase (AN7418) orthologous gene is not present in the genomes of many aspergilli, including A. terreus. In addition, the identity of salicylate 5-monooxygenase is even more obscure as it has not been previously reported in fungi. As observed here for A. nidulans, 32 aromatic-ring FAD-binding monooxygenases (IPR002938) were found moderately to highly expressed and upregulated, including six phenol monooxygenases (IPR038220), hindering a precise assignment of the salicylate monooxygenases at this stage.

The RNA-seq data showed the upregulation of several genes of the phenylalanine, tyrosine, and tryptophan catabolism, as well as three gene clusters, namely, the homogentisate cluster (ATEG_05922 to ATEG_05926), the nicotinate-inducible cluster (ATEG_08614 to ATEG_08620 and ATEG_05673), and a cluster unknown in fungi (Fig. 3; Table S5). The first two have been previously described in A. nidulans (24, 25). The last cluster is here described for the first time in fungi, which we designate as the 3-hydroxyanthranilate catabolic cluster. Three of the comprised genes of this cluster putatively encode three consecutive enzymes of the tryptophan kynurenine pathway (26), namely, 3-hydroxyanthranilate dioxygenase (3HAO; ATEG_10009-10; not annotated in the public databases [see Table S2 in the supplemental material]), aminomuconate semialdehyde dehydrogenase (AMSDH; ATEG_10010), and 2-amino 3-carboxymuconate 6-semialdehyde decarboxylase (ACMSD; ATEG_10009). This cluster, as well as some upstream pathway genes, was observed to be upregulated in salicylate (Fig. 3; Table S5). Three types of indoleamine 2,3-dioxygenases (IDOs) present in aspergilli can catalyze l-tryptophan to kynurenine at the start of the pathway, although with different biochemical characteristics and unknown functional roles (27). IDOα (ATEG_03482) was found downregulated and highly expressed, while IDOβ (ATEG_00759) and IDOγ (ATEG_01829) were found upregulated and with even higher expression levels. However, a fourth IDO gene (ATEG_07359) is present in the genome of A. terreus, which exhibited almost no expression in salicylate and acetate media. The 3-hydroxyanthranilate dioxygenase genes in fungi have been merely associated with an alternative path for NAD^+^ biosynthesis (28). On the contrary, our data support the existence of the catabolic tryptophan kynurenine pathway forming 2-aminomuconate in fungi (Fig. 3 and 4), regardless of the fact that none of the intermediates of this pathway (e.g., 3-hydroxyanthranilate) could be definitely identified (Fig. S5). Under the cultivation conditions used here, also catechol did not accumulate, hindering its detection, similar to what was reported before (9). The reaction product of 3-hydroxyanthranilate dioxygenase is 2-amino 3-carboxymuconate semialdehyde, which can be converted enzymatically to 2-aminomuconate semialdehyde or decay nonenzymatically to quinolinate, which is the precursor of NAD^+^ (Fig. 3). Finally, 2-aminomuconate semialdehyde can also decay nonenzymatically to picolinate, a bidentate chelating agent, of which the metabolism remains largely unknown (26).

**FIG 5:**
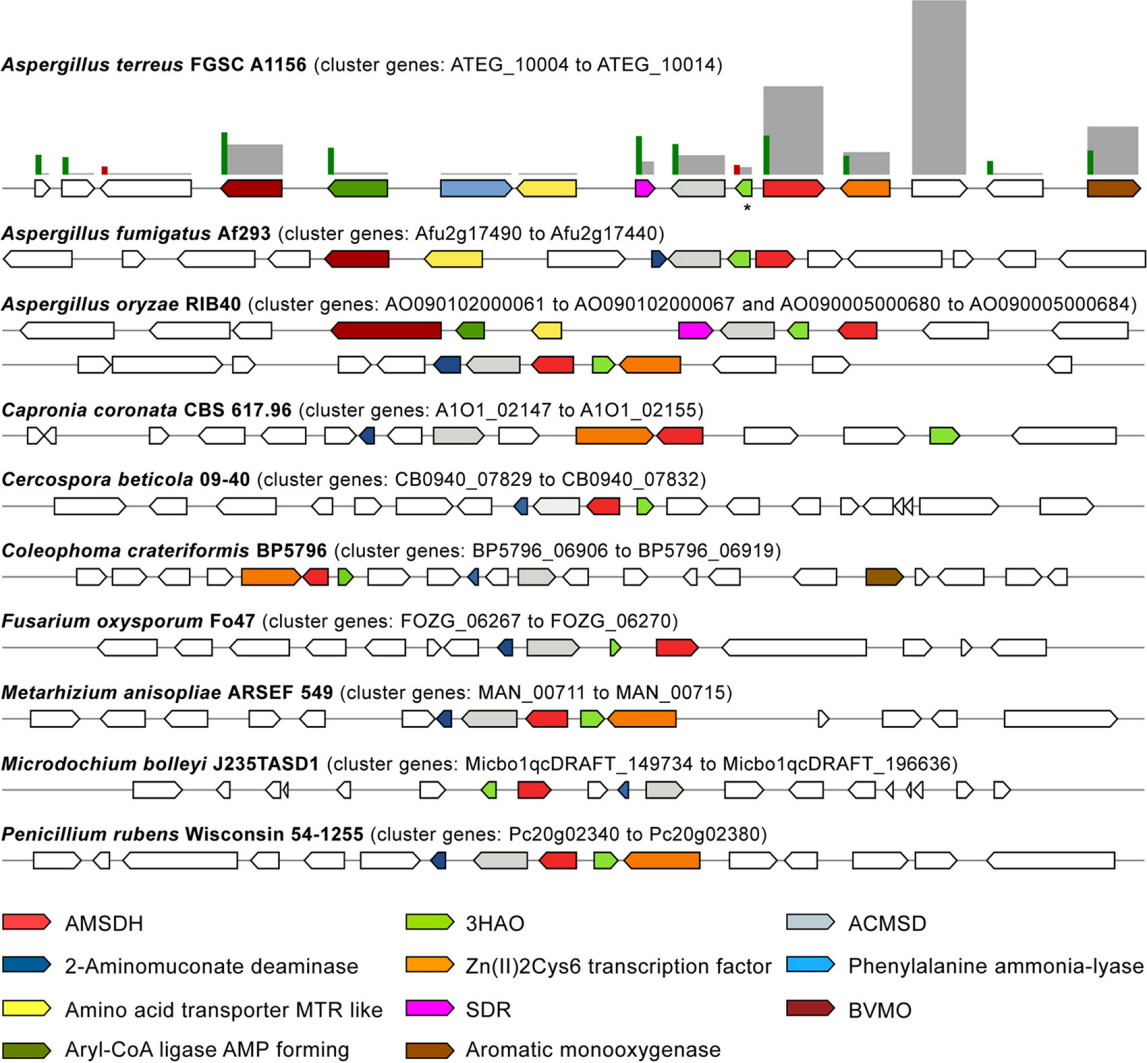
Representation of the 3-hydroxyanthranilate catabolic gene cluster in some *Ascomycota*. The cluster includes genes coding for 3-hydroxyanthranilate-3,4-dioxygenase (3HAO), 2-amino-3-carboxymuconate-6-semialdehyde decarboxylase (ACMSD), 2-aminomuconate semialdehyde dehydrogenase (AMSDH), and a novel putative 2-aminomuconate deaminase (AMDA; 2-hydroxymuconate-forming), except in A. terreus, that does not contain the last gene. Some *Aspergillus* syntenic genes, namely, a Baeyer-Villiger monooxygenase (BVMO), a short-chain dehydrogenase/reductase (SDR) and an amino acid transporter, are also represented. Clusters were identified within annotated genomes through sequence homology searches (MultiGeneBlast software). Genes within the cluster are represented using a color code and an asterisk indicates a nonannotated gene. The regulation and abundance of the differentially expressed genes (RNA-seq) are represented by bars of different scales and colors (upregulated in green, downregulated in red, and RPKM in gray).

The 3-hydroxyanthranilate catabolic gene cluster is present across *Ascomycota*, including some species of *Aspergillus*, *Fusarium*, Metarhizium, and *Penicillium* (Fig. 5), but absent in *Saccharomycotina*. In some aspergilli, two similar clusters are present (e.g., in Aspergillus oryzae), with the second cluster showing structural conservation and higher sequence homology to the cluster found in Metarhizium and *Penicillium*; indicative of a more recent horizontal gene transfer event. The cluster is absent in A. niger and Fusarium graminearum, where l-tryptophan catabolism proceeds via anthranilate, 2,3-dihydroxybenzoate, and the catechol branch of the 3-oxoadipate pathway (29, 30). A fourth gene of the YjgF/YER057c/UK114 family (IPR006175) is recurrently present in the cluster, but not in A. terreus (Fig. 5). Proteins of this diverse family are expected to have various metabolic roles extending from endoribonuclease activity and 2-aminomuconate deaminase to enamine/imine deaminase of the branched-chain amino acid biosynthesis pathway (31). Its orthologous gene in A. terreus (ATEG_05745) was found moderately expressed and upregulated in salicylate (Fig. 3 and 4): hence, possibly it encodes a 2-aminomuconate deaminase. It has been shown that this deamination reaction may also occur nonenzymatically, forming the same products: 2-hydroxymuconate and its tautomer 2-oxo-3-hexenedioate (32). In line with this, herein we noticed that a putative 4-oxalocrotonate tautomerase gene (ATEG_05150) was found upregulated, suggestive that the encoded protein (IPR014347) may mediate 2-oxo-3-hexenedioate formation (Fig. 3). This compound may undergo further reduction to 2-oxoadipate or decarboxylation and hydration to 4-hydroxy-2-oxopentanoate. The subsequent decarboxylation of 2-oxoadipate to glutaryl-CoA by the oxoglutarate dehydrogenase complex (encoded by ATEG_03911 and ATEG_09000) could not be verified by the RNA-seq data. We observed that the TCA cycle in A. terreus (and in A. nidulans) was found highly downregulated in salicylate medium, and the predicted glutaryl-CoA dehydrogenase (ATEG_08866) was also found downregulated (Table S5).

Several genes of the leucine catabolic pathway were found extremely expressed and upregulated in salicylate, namely, the E1 and E2 components of the branched-chain α-keto acid dehydrogenase complex (ATEG_03862, ATEG_06635, and ATEG_09087), isovaleryl-CoA dehydrogenase (ATEG_06574), and 3-methylcrotonyl-CoA carboxylase (ATEG_06573 and ATEG_06576). However, the remaining genes of the leucine catabolism were downregulated or only moderately expressed (Table S5). Taken together, it remains unclear if those genes that underwent upregulation have a role in the catabolism of the branched-chain 2-oxoacid 4-hydroxy-2-oxopentanoate, namely, in its decarboxylation to 3-hydroxybutyryl-CoA. The predicted 3-hydroxybutyryl-CoA dehydrogenase (ATEG_02736) and the downstream acetyl-CoA acetyltransferase (ATEG_00012) were found downregulated, although highly expressed (Fig. 3). Importantly, the catabolism of branched-chain amino acids was previously shown to be upregulated under conditions where 2-hydroxymuconate was produced (33).

In aspergilli, the 3-hydroxyanthranilate catabolic gene cluster shows synteny to genes likely coding in the peripheral pathways (Fig. 5), some of which upregulated by salicylate (ATEG_10004, ATEG_10005, and ATEG_10008). Two of these genes, encoding an aryl-CoA ligase (AMP forming) (ATEG_10005) and an SDR (ATEG_10008), show low homology to characterized proteins. However, the FAD-binding monooxygenase ATEG_10004 gene, which here underwent major expression, contains the sequence motif of a Baeyer-Villiger monooxygenase (34), a distinct class of flavoproteins that catalyze the insertion of an oxygen atom in a C–C bond of a ketone forming an ester. In addition, another FAD-binding monooxygenase gene (ATEG_10014) that was found greatly expressed and upregulated shows discrete homology to the salicylate 1-monooxygenase (decarboxylating) gene, which is located in the vicinity of the 3-hydroxyanthranilate catabolic cluster (Fig. 5).

**FIG 6:**
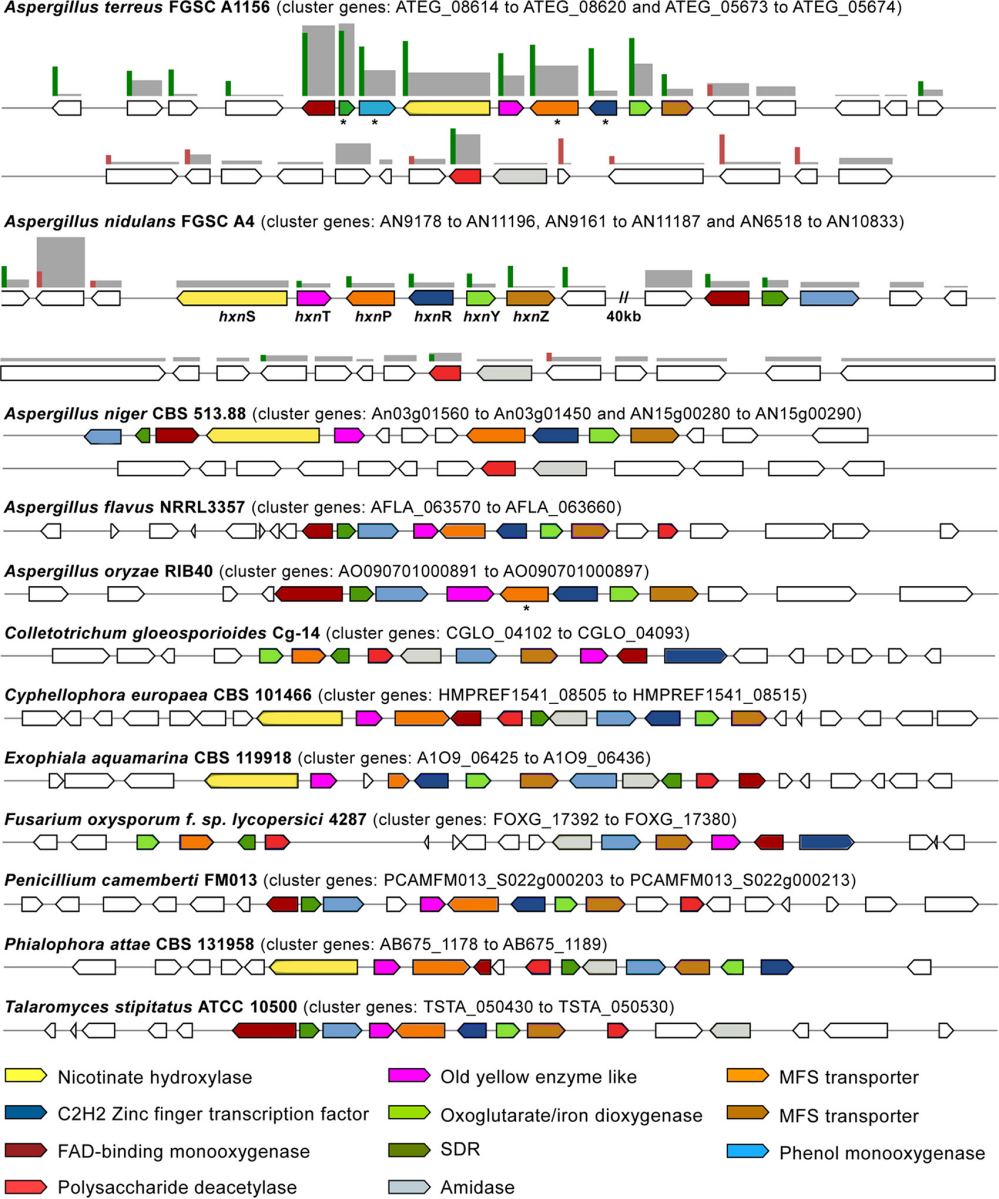
Representation of the nicotinate gene cluster in *Ascomycota*. The cluster includes the following genes: nicotinate hydroxylase (*hxnS*) and the remaining five nicotinate-inducible orthologous genes of Aspergillus nidulans (*hxnT*, *hxnP*, *hxnR*, *hxnY*, and *hxnZ*) as well as five additional genes that are recurrently clustered in *Ascomycota* genomes. Clusters were identified within annotated genomes through sequence homology searches (MultiGeneBlast software). Genes within the cluster are represented using a color code, and an asterisk indicates a poorly annotated or nonannotated gene. A double slash indicates a gap within the same chromosome. The differentially expressed genes (RNA-seq) regulation and abundance is represented by bars of different scales and color (upregulated in green, downregulated in red, and RPKM in gray).

The six orthologous genes of the A. nidulans nicotinate-inducible gene cluster (25) are part of a highly expressed and upregulated gene cluster of nine genes in A. terreus grown in salicylate that includes three genes not yet described: two FAD-dependent monooxygenase genes (ATEG_08614 and ATEG_08615b) and an SDR gene (ATEG_08615a) (Fig. 6). ATEG_08614 shows discrete homology to the bacterial 6-hydroxynicotinate 3-monooxygenase (Q88FY2) that forms 2,5-dihydroxypyridine as part of nicotinate catabolism, but also to 3-hydroxybenzoate 6-monooxygenase (Q9F131) and orsellinic acid 1-monooxygenase (J4VWM7). ATEG_08615b is a phenol hydroxylase (IPR012941). Their major coregulation in salicylate is suggestive of participation in the nicotinate metabolism. In addition, its cluster preservation in aspergilli and across *Ascomycota*, being however divided into two clusters in the genome of A. nidulans (Fig. 6), strengthens the hypothesis of their role in the nicotinate metabolism. Expanding the cluster analysis across numerous fungal species (Fig. 6) revealed the presence of two additional colocalized genes—a deacetylase gene (ATEG_05673) and an amidase gene (ATEG_05674)—that are not located in the nicotinate-inducible cluster in the majority of aspergilli, including in A. terreus and A. nidulans. In salicylate, only the deacetylase gene (ATEG_05673) was found highly expressed and upregulated, consistent with its assignment to the nicotinate metabolism. In general, the nicotinate-inducible genes of A. nidulans showed to be somehow upregulated in salicylate medium, although to a much lesser extent compared to A. terreus (Fig. 6). This together with the moderate co-upregulation of the single-copy 3-hydroxyanthranilate dioxygenase (3HAO) gene (AN11252, orthologous to ATEG_00088) in A. nidulans (Table S4) pushes forward the idea of an alternative pathway for the metabolism of aromatic compounds. The existence of such a metabolic route possibly explains why the metabolism of the quorum-sensing molecule phenylethyl alcohol yielded the pyridine ring of NADH and NADPH in Candida albicans (35).

The gentisate-like cluster contains a FAD-binding monooxygenase gene (ATEG_06711) that did not undergo initial upregulation (Fig. 4). A similar trend was noticed for the gentisate 1,2-dioxygenase orthologous gene (ATEG_06712). These observations exclude their foreseen involvement in the hydroxylation of salicylate to gentisate and its subsequent ring cleavage. We have identified another gentisate 1,2-dioxygenase gene (ATEG_07116-17), which is not currently annotated (Table S2), that showed no altered expression levels during cultivation in salicylate (Fig. 4). Both A. terreus and A. nidulans were unable to grow at the expense of gentisate (5 or 10 mM), regardless of the capacity to cometabolize gentisate (depletion of 5 mM in less than 3 days with acetate-pregrown cultures). In addition, gentisate may also be transformed to hydroxyquinol by salicylate 1-monooxygenase (36). However, the expression analysis of the two hydroxyquinol 1,2-dioxygenase genes (ATEG_00629 and ATEG_07465), orthologous of the two recently biochemically characterized genes of A. niger (11), as well as of the predicted maleylacetate reductase gene (ATEG_03722), showed no great alteration (Fig. 4; Table S5). The mild upregulation of the hydroxyquinol pathway is more likely to be related to the detection of hydroquinone (Fig. S5), the latter resulting from non-oxidative decarboxylation of gentisate (Fig. 3).

The homogentisate pathway for the catabolism of phenylalanine and tyrosine (chorismate as a common precursor) was found mostly upregulated in salicylate (Fig. 3 and 4). Several routes can be used for the metabolism of their corresponding precursors, phenylpyruvate and 4-hydroxyphenylpyruvate, likely involving a small group of enzymes capable of mediating analogous reactions (37). In line with this idea, we observed that a pyruvate decarboxylase PDC1-like gene (ATEG_06881) and several alcohol and aldehyde dehydrogenase genes (Table S5), potentially coding for enzymes mediating the production of fusel alcohols and acids (e.g., tryptophan to tryptophol) (38), underwent upregulation in salicylate. These observations suggest that the Ehrlich pathway was likely upregulated, further reinforced by the concurrent upregulation of phenylacetate 2-hydroxylase and 3-hydroxyphenylacetate 6-hydroxylase orthologous genes (ATEG_09785 and ATEG_08499) encoding monooxygenases of the fusel acids phenylacetate and 4-hydroxyphenylacetate or their products.

### Conclusions.

Analysis of the transcriptome associated with the catabolism of salicylate in two species of *Aspergillus* showed its intimate relationship with the secondary metabolism, which was particularly obvious for metabolites requiring action of aromatic prenyltransferases. Exogenous aromatic compounds and their metabolites can provide the building blocks for the synthesis of many secondary metabolites, as well established, e.g., for penicillin (i.e., phenylacetate) (37). We observed that the metabolism of salicylate yields gentisate in A. terreus, likely also producing gentisyl alcohol, an intermediate of secondary metabolites such as patulin (39, 40). This observation suggests that usage of salicylate (or gentisate) as a carbon source may improve the production of target metabolites. Salicylate also upregulated several other catabolic pathways in A. terreus, including the tyrosine metabolism that may provide plentiful SM′ precursors. Tyrosine catabolic genes and secondary metabolite core biosynthetic genes showed similar regulation in penicillia (41). Understanding the relationship between salicylate and SM in fungi may provide unexpected findings on plant-fungus interaction, specifically how fungi counteract salicylate-dependent plant defense mechanisms (42).

Collectively our data showed the high complexity of the metabolism of salicylate in A. terreus with concomitant upregulation of several pathways for the catabolism of aromatic compounds at distinct timings (Fig. 4). On the contrary, A. nidulans used only a major route (Fig. S2), similar to that previously reported for many fungal species (see reference 9 and references therein). One unexpected idiosyncrasy of A. terreus is the formation of significant amounts of gentisate in the salicylate medium, and its subsequent metabolism through pathways not yet fully disclosed. However, it is clear that gentisate metabolism led to upregulation of the homogentisate pathway, as well as that of the nicotinate catabolism and 3-hydroxyanthranilate catabolic pathways, none of which could be anticipated. This idea is strengthened because in A. nidulans, which did not yield gentisate through salicylate catabolism, these pathways remained mostly unregulated (or some are even absent). The joint action of the nicotinate metabolism and the 3-hydroxyanthranilate catabolic pathway in A. terreus should be seen as a novel route for the degradation of aromatic compounds. One of the most striking observations is that the gentisate-like gene cluster in this fungus is apparently not involved in the catabolism of the formed gentisate, suggestive of a yet unknown role. In addition, 2-hydroxymuconate is one possible intermediate of the 3-hydroxyanthranilate catabolic pathway, as observed elsewhere in pyrogallol catabolism (43), possibly denoting a convergence of the two pathways. Naturally, this may provide a straightforward strategy for solving the assignment of the genes associated with the catabolism of 2-hydroxymuconate in fungi.

To date, the catabolism of tryptophan in fungi was thought to involve anthranilate and the catechol branch of the 3-oxoadipate pathway. On the contrary, our data on A. terreus and the presence of the 3-hydroxyanthranilate catabolic pathway genes in some *Ascomycota* genomes suggest that the tryptophan kynurenine pathway in these species shares similarity with that of higher eukaryotes. This opens the exciting possibility of using fungi as model organisms for the investigation of diseases related to tryptophan catabolism: e.g., study of the toxic effects of quinolinate in the progress of Huntington´s disease (26). The 3-hydroxyanthranilate catabolic pathway shows lower occurrence in *Ascomycota* genomes than the other central aromatic catabolic pathways. Why a smaller number of *Ascomycota* possess this catabolic plasticity raises intriguing questions about its role and evolutionary traits, especially as some aspergilli have two similar 3-hydroxyanthranilate catabolic gene clusters.

Finally, the sustainable bio-based production of organic acids requires the usage of renewable nonfood biomass, with lignin moving toward the pole position. The transformation of lignin-related aromatic monomers into value-added chemicals may be conceived by the so-called “biological funneling” (5). To date, a delocalized cytosol biosynthesis of TCA cycle intermediates (3) has been used for their production from sugars. Since the aromatic hydrocarbon catabolism in fungi occurs also at the cytosol (44, 45), it is possible to foresee engineered fungal strains as tools to convert these compounds into central intermediates of the catabolism of aromatics and subsequently into organic acids. Our study provides another piece of the puzzle of the catabolism of aromatics in fungi, revealing new catabolic paths (some unexpected) and their putative genes, and proposes future paths to further dissect their functional roles, evolutionary traits, and technological relevance.

## MATERIALS AND METHODS

### Strains and growth conditions.

Aspergillus nidulans FGSC A4 or A1147 and A. terreus FGSC A1156 asexual spores were harvested and maintained as frozen suspensions at –80°C (46). Cultures were initiated with 10^6^ spores/ml and incubated with orbital agitation (250 rpm) in the dark at 37°C. Batch cultivations were performed in 250-ml Erlenmeyer flasks with a working volume of 50 ml. A low-nitrogen minimal medium was used containing per liter 3 g NaNO_3_, 0.01 g ZnSO_4_·7H_2_O, 0.005 g CuSO_4_·5H_2_O, 0.5 g MgSO_4_·7H_2_O, 0.01 g FeSO_4_·7H_2_O, and 0.5 g KCl. Filter-sterilized salts were added to an autoclave-sterilized 100 mM potassium phosphate (pH 6.0) solution. The carbon sources were added either directly to the phosphate solution for sterilization (60 mM sodium acetate [control]) or to the mineral medium after filter sterilization (20 mM sodium salicylate). Solid minimal medium was jellified with 5 g/liter agarose, and cultures were grown at 30°C in the dark, on top of a polyvinylidene difluoride (PVDF) membrane (Hybond-P, 0.45-μm pore; GE Healthcare) (47).

### Metabolite identification and quantification.

Filtrated culture media and the ensuing ethyl acetate extracts (1:1 [vol/vol]; ca. 50-fold concentrated) were chromatographically analyzed for the identification and/or quantification of the aromatic compounds and their putative intermediates either by ultraperformance liquid chromatography (UPLC) (48) or by high-performance liquid chromatography (HPLC). HPLC was performed using a Symmetry C_18_ column, 5 μm, 4.6 mm by 250 mm (Waters Corporation, USA), set at 26°C. The mobile phase, at a flow rate of 0.9 ml min^−1^, consisted of 0.1% trifluoroacetic acid (solvent A) and acetonitrile (solvent B), set as follows: a linear gradient of 0.5 to 100% B in 30 min, followed by 10 min at 100% B, with 2 min to return to the initial conditions and 10 min to reequilibrate the column. To guide the chromatographic identifications, the following standards were used: salicylic acid, 2,3-dihydroxybenzoic acid, gentisic acid, catechol, anthranilic acid, 3-hydroxyanthranilic acid, and *cis*,*trans*-muconic acid. The ethyl acetate extracts were also analyzed by gas chromatography-time of flight mass spectrometry (GC-TOF MS) with a Pegasus BT mass spectrometer equipped with an L-PAL3 automatic sampler (LECO). The system was operated using the ChromaTOF software (v5.40.12.0.60635) for Pegasus BT. The separation of the analytes was carried out using a DB-5MS Ultra Inert capillary column (25 m by 0.250 mm by 0.24 μm; Agilent). The carrier gas used was helium at a flow rate of 1.0 ml/min. The split ratio for the injector was set to 1Å:Å5, with a total injection volume of 1 μl. The front inlet and ion source were both kept at 250°C and the transfer line at 290°C. Oven temperature was set to equilibrate at 60°C for 1 min, before initiation of sample injection and GC run. Following sample injection, oven temperature was maintained at 60°C for another 1 min, increased at a rate of 5°C/min to 290°C, and held for 5 min. The MS detection was operated in electron ionization (EI) mode (70 eV) with a detector voltage of 1,800 V. Full scan mode with a mass range of *m*/*z* 40 to 600 and acquisition rate of 15 Hz was used as data acquisition method. Acquisition delay was set at 420 s. Chromatogram data acquisition, baseline correction, peak deconvolution, analyte alignment, peak area integration, and analyte identification by mass spectral searches (based on National Institute of Standards and Technology and Wiley libraries) were performed using the LECO ChromaTOF. Peaks with a similarity of 80% or more and a maximum relative Kovats index deviation of 10% were assigned putative by-product identities based on the mass spectral libraries. The analyses focused on those peaks present in all the salicylate-derived extracts but absent in the control (acetate medium ethyl acetate extracts), relying on the spectral features of pure standards whenever possible.

### Transcriptome analysis.

Total RNA extraction of liquid-grown mycelia was performed essentially as previously described (46), using a Tissuelyser LT (Qiagen) for cell disruption. The quality and quantity of RNA were determined by capillary electrophoresis using the HS RNA kit and 5200 Fragment analyzer (Agilent Technologies). For single-end RNA sequencing (RNA-seq), libraries were generated using the Smart-Seq2 mRNA assay (Illumina, Inc.) according to the manufacturer’s instructions. Twelve samples were indexed and sequenced on the Illumina NextSeq550 (20 million reads per sample). Generated FastQ files were analyzed with FastQC, and any low-quality reads were trimmed with Trimmomatic (49). All libraries were aligned to the corresponding model fungus, either A. nidulans FGSC A4 genome assembly (ASM14920.v2) or A. terreus NIH2624 genome assembly (ASM14961.v1) with gene annotations from Ensembl Fungi v.37 using HISAT2 v.2.1.0 (50) and only matches with the best score were reported for each read. All RNA-seq experiments were carried out in three biological replicates. Differential expression analysis was performed using DESeq2 v.1.24.0 (51). The principal-component analysis (PCA) plot and MA plots were also generated using the DESeq2 package. The genes that showed more than log_2_ 1-fold expression changes with an adjusted *P* value of <0.05 are defined as significantly differentially expressed genes in this analysis. Transcript abundance was defined as the number of reads per kilobase of transcript per million mapped reads (RPKM). The full genomes were scanned using InterProScan v.5.32-71.0 (52) to build a protein domain database for further enrichment analyses. The enrichment analyses were performed with the FunRich software v.3.1.3 (53), using the constructed protein domain databases as the terms library and the hypergeometric test with a *P* value of <0.05.

### Reverse transcription-quantitative PCR analysis.

Total RNA extraction was performed as reported above, and cDNA synthesis was performed as previously described (46). Oligonucleotide pairs were designed using Primer-BLAST (54) and supplied by STAB Vida (Oeiras, Portugal) (see Table S1 in the supplemental material). The reverse transcription-quantitative PCR (RT-qPCR) analysis was performed in a CFX96 thermal cycler (Bio-Rad), using the SsoFast EvaGreen Supermix (Bio-Rad), 250 nM each oligonucleotide, and the cDNA template equivalent to 10 ng of total RNA, at a final volume of 10 μl per well, in three biological replicates. The PCR conditions were enzyme activation at 95°C for 30 s; 40 cycles of denaturation at 95°C for 5 s and annealing/extension at 60°C for 15 s, and melting curve obtained from 65 to 95°C, consisting of 0.5°C increments for 5 s. Data analyses were performed using the CFX Manager software v.3.1 (Bio-Rad). The expression of each gene was taken as the relative expression in pairwise comparisons of each condition relative to the acetate control. The expression of all target genes was normalized by the expression of the 60S ribosomal protein L33-A gene, AN2980 or ATEG_01624.

10.1128/mSystems.00230-20.6 TABLE S1 List of oligonucleotides used in RT-qPCR. Download Table S1, PDF file, 0.5 MB. Copyright © 2021 Martins et al. 2021 Martins et al. This content is distributed under the terms of the Creative Commons Attribution 4.0 International license.

### Gene structure prediction.

Automated gene structure prediction of new genes or of poorly annotated genes was done using FGENESH with or without similar protein-based gene prediction (55). Gene structure was manually curated (Table S2) and used in the transcriptome analysis.

10.1128/mSystems.00230-20.7 TABLE S2 Gene sequences of predicted new genes or of poorly annotated genes. Download Table S2, PDF file, 0.5 MB. Copyright © 2021 Martins et al. 2021 Martins et al. This content is distributed under the terms of the Creative Commons Attribution 4.0 International license.

### Identification of gene clusters.

Metabolic gene clusters were identified within annotated genomes through sequence homology searches using MultiGeneBlast v.1.1.13 (56). A recent customized database created from online GenBank entries was done using the same software. Identification of secondary metabolite biosynthesis gene clusters was done using antiSMASH v.5.0 (57), and the results were incorporated into available information (see Table S3 in the supplemental material), namely, from precomputed results at MycoCosm obtained by using SMURF (58, 59).

10.1128/mSystems.00230-20.8 TABLE S3 Secondary metabolite gene clusters of Aspergillus nidulans and Aspergillus terreus. Boundaries were manually curated from data obtained in the literature and by using antiSMASH or from JGI MycoCosm. Download Table S3, PDF file, 0.5 MB. Copyright © 2021 Martins et al. 2021 Martins et al. This content is distributed under the terms of the Creative Commons Attribution 4.0 International license.

### Data availability.

Transcript RPKM values of differentially expressed genes are contained in Tables S4 and S5, and raw data with metadata were deposited in the Sequence Read Archive accession no. PRJNA612036.

10.1128/mSystems.00230-20.9 TABLE S4 Aspergillus nidulans RNA-seq data of differentially expressed genes. Emphasis is given to expression levels of genes of the catechol branch of the 3-oxoadipate pathway (and of other relevant pathways) in response to salicylate. Download Table S4, XLSX file, 0.8 MB. Copyright © 2021 Martins et al. 2021 Martins et al. This content is distributed under the terms of the Creative Commons Attribution 4.0 International license.

10.1128/mSystems.00230-20.10 TABLE S5 Aspergillus terreus RNA-seq data of differentially expressed genes. Emphasis is given to expression levels of genes of the catechol branch of the 3-oxoadipate pathway, the presumed gentisate pathway, the metabolism of acetate, phenylalanine, tyrosine, and tryptophan, as well as two other relevant gene clusters, in response to salicylate. Download Table S5, XLSX file, 1.0 MB. Copyright © 2021 Martins et al. 2021 Martins et al. This content is distributed under the terms of the Creative Commons Attribution 4.0 International license.
