# Supplementary figures and images for: Twists and Turns in the Salicylate Catabolism of Aspergillus terreus, Revealing New Roles of the 3-Hydroxyanthranilate Pathway
Source: mSystems. 2021 Jan 26;6(1):e00230-20. doi: 10.1128/mSystems.00230-20 (PMC7842363; doi:10.1128/mSystems.00230-20)

**A**

## MA Plot

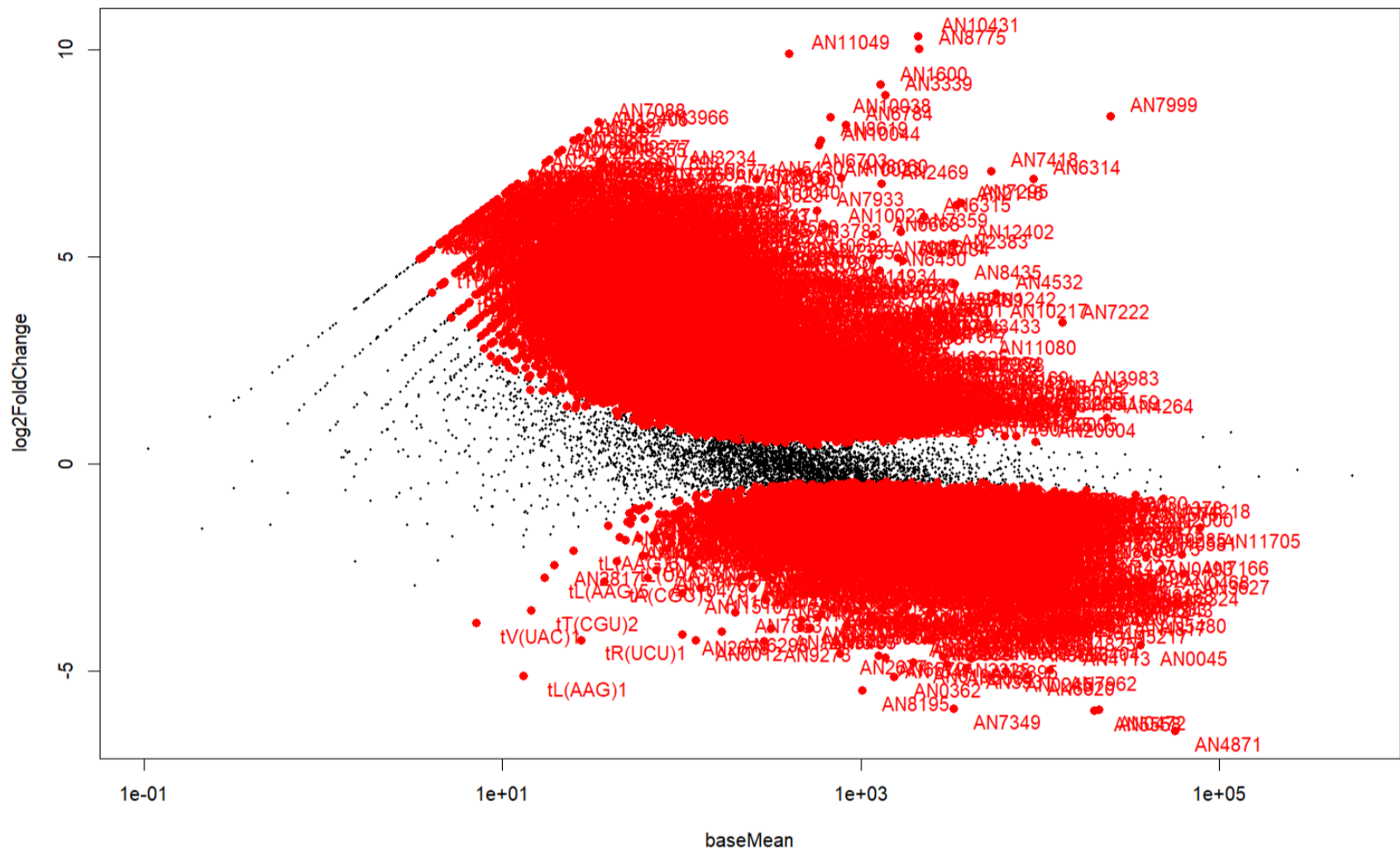

# B

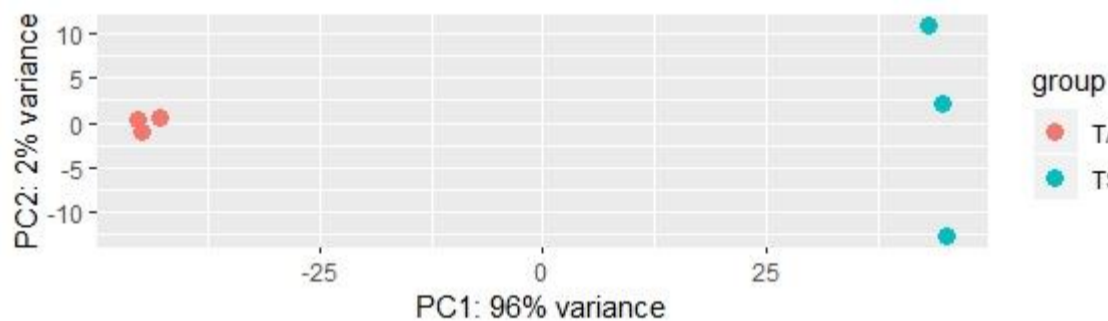

## MA Plot

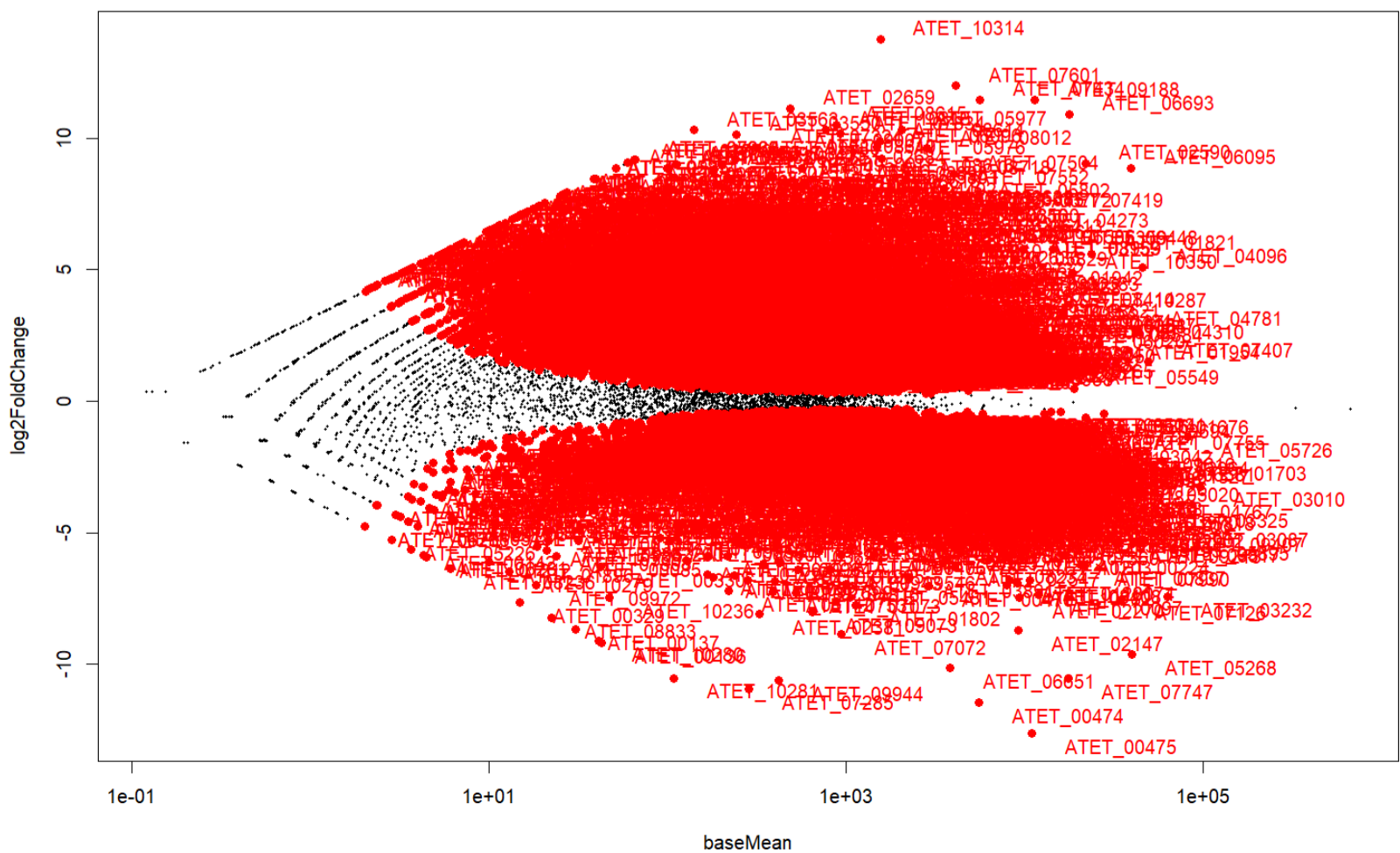

Supplement: FIG S1 [file mSystems.00230-20-sf001.pdf]

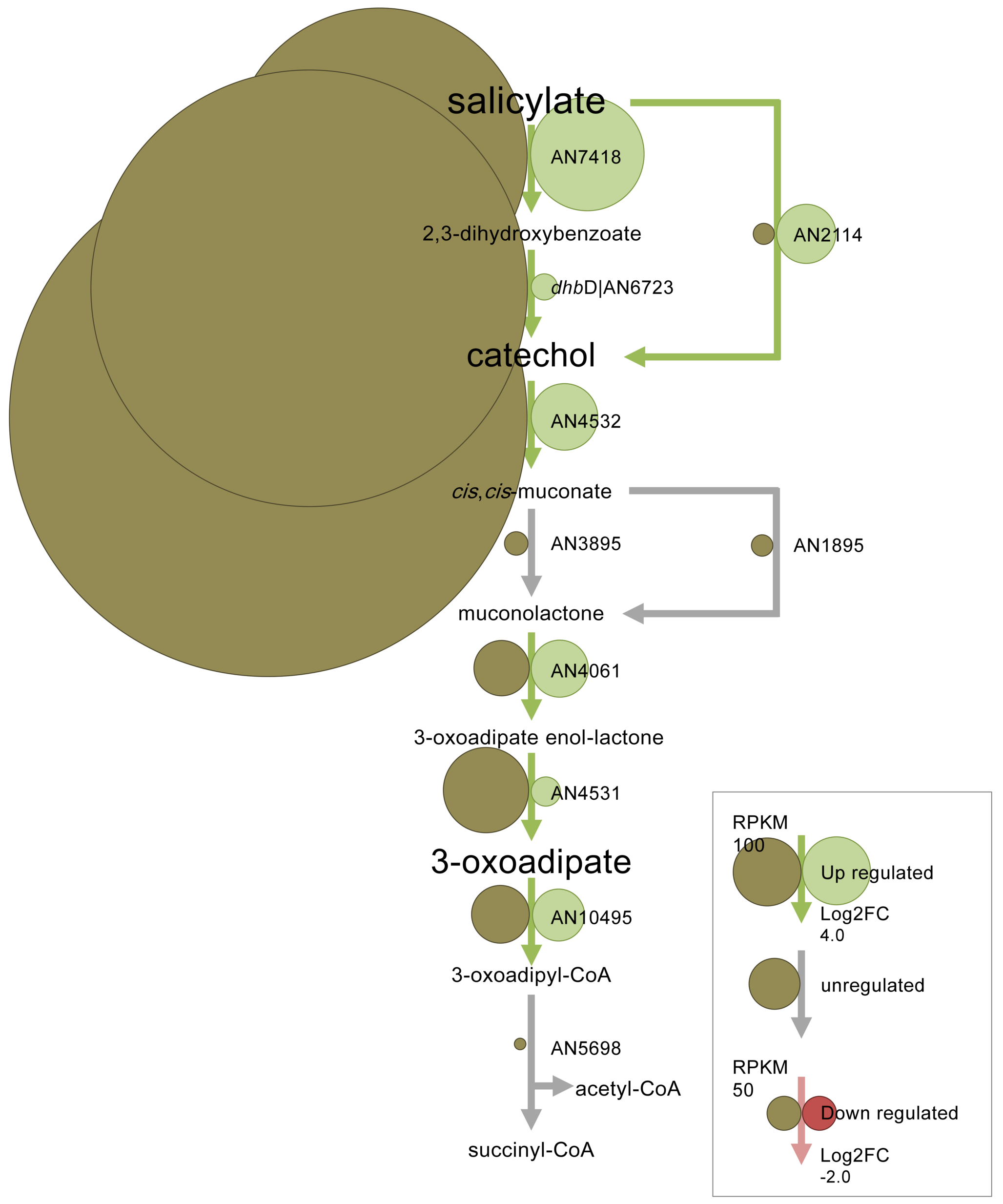

Supplement: FIG S2 [file mSystems.00230-20-sf002.tif]

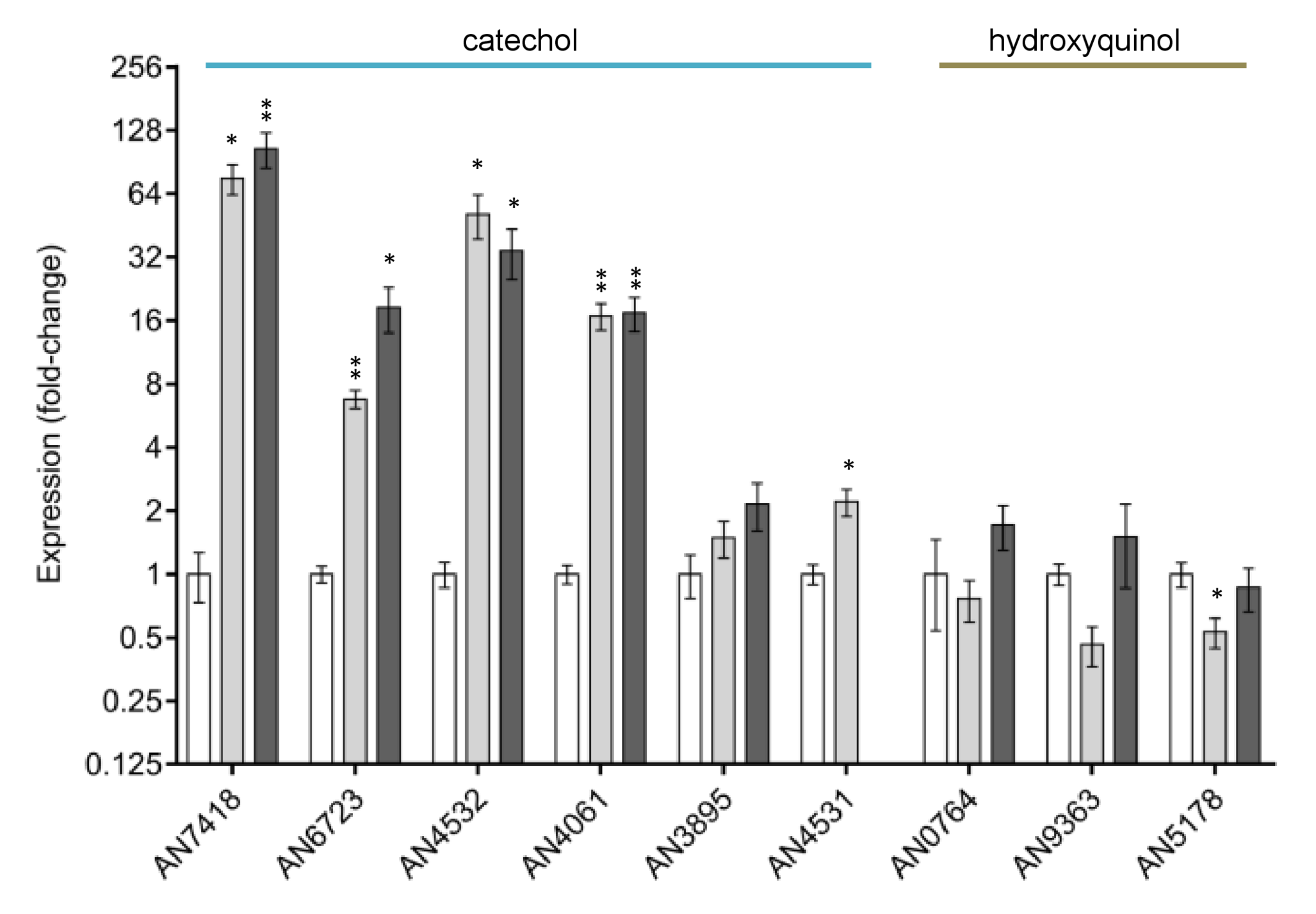

Supplement: FIG S3 [file mSystems.00230-20-sf003.tif]

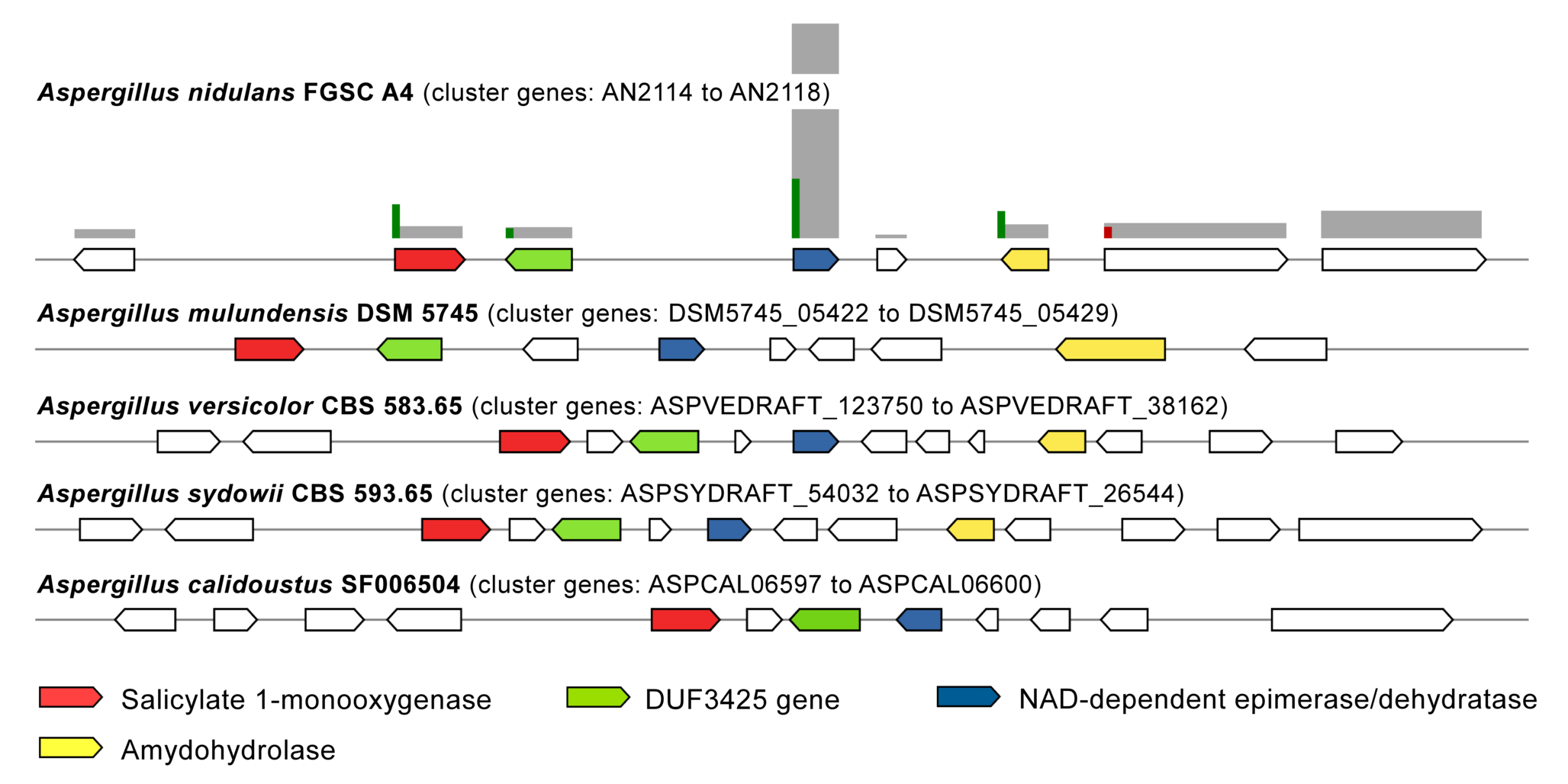

Supplement: FIG S4 [file mSystems.00230-20-sf004.tif]

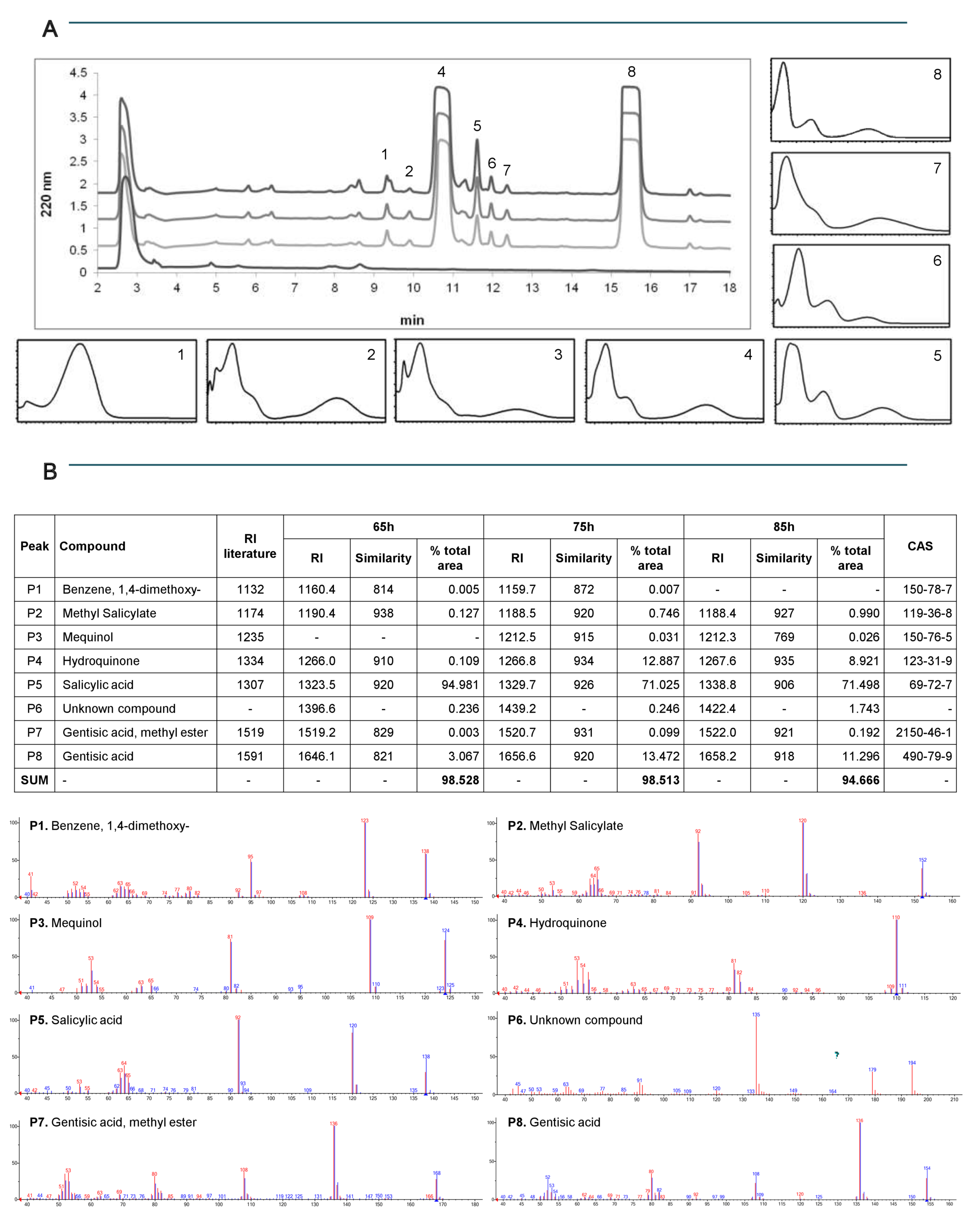

Supplement: FIG S5 [file mSystems.00230-20-sf005.tif]
