# Supplementary material for: Twists and Turns in the Salicylate Catabolism of Aspergillus terreus, Revealing New Roles of the 3-Hydroxyanthranilate Pathway
Source: mSystems. 2021 Jan 26;6(1):e00230-20. doi: 10.1128/mSystems.00230-20 (PMC7842363; doi:10.1128/mSystems.00230-20)
Supplement: TABLE S1 [file mSystems.00230-20-st001.pdf]

Table S1. List of oligonucleotides used in RT-*q* PCR.

Gene name	Description	Forward sequence 5'-3'	Reverse sequence 5'-3'
AN0764	hydroxyquinol 1,2-dioxygenase	CGAAGCCGAGGAACTGTCTC	CCGTTGAGTCGGTTTCCCAA
AN2980	60S ribosomal protein L33-A	TCTACCGGGCTAAGCGTGA	CACGGACAGTAGCACCGAA
AN3895	muconate cycloisomerase	TGCAGACGTCCTCGTCGA	CCCCTTCGTAGCCAAAGCA
AN4061	muconolactone isomerase	ATGCTCTACGGGACGGTCA	AGACGGATCCAGATAAACCTGA
AN4531	3-oxoadipic enol-lactone hydrolase	GCAGATCGCGTGAAGGGTA	GCCAGTAGCTTGGTAGGGAA
AN4532	catechol 1,2-dioxygenase	GTCAATGCCATGGGCGAGA	GTACTCCGGCCATCCACTC
AN5178	maleylacetate reductase	TGGTCTGGGGAAGGCTATCA	CGGGGAGGATCTTGGGATCA
AN6723	*dhb* D, 2,3-dihydroxybenzoate carboxylyase	ACCGTAAATGGCTCGTTGGA	GAAGGGTATGTGCTCGCCTA
AN7418	salicylate 1-monooxygenase	GCGCTGTTGTTGATGATGGA	CATCGCAACCGACGAGGAA
AN9363	hydroxyquinol 1,2-dioxygenase, putative	GCGACCCTTACGAGACATCC	AAGCACAAAGTCGTGCCTCA
ATEG_00088	3-hydroxyanthranilate 3,4-dioxygenase	GTTCCAGGACATCCCCATCC	CCAGAGCATGGTGTCAGTGG
ATEG_00629	hydroxyquinol 1,2-dioxygenase	GCGTCTTCTGGTTCAAGGCT	GATCGAACCCCTCCTTCTGG
ATEG_01624	60S ribosomal protein L33-A	AGGTTCGGGGTTCCAACATC	TGGAGGGGTAGAGCATGACG
ATEG_03095	muconolactone isomerase	GGAACAGTTCTACGCCACCA	CCATGACGGAGGGCTAGGAT
ATEG_03722	maleylacetate reductase	ACTCGCTCCGACCCTAAGAT	TAGTGTTGCGCGCATAGAGG
ATEG_05745	2-aminomuconate deaminase	ACAAGGGCATGAACGACGAA	CACCACTCACAAATCGCAGTG
ATEG_05923	homogentisate 1,2-dioxygenase	CACTGCCATTGCCGACTTTG	CCACCAGTCTTCGCATCGTA
ATEG_05926	homogentisate cluster TF	CGCTTTGAACATTGACGCGA	ATGAGCTCGTAATCCGCAGG
ATEG_06350	2,3-dihydroxybenzoate carboxy-lyase	CCTTTGACTTGTGGCGCATC	GAGTGGTGGTGGAGAAGTGG
ATEG_06711	gentisate cluster aromatic monooxygenase	CTTTGCTGCCTACAGAGCCA	TGCCTGCACCGATTGTGTAT
ATEG_06712	gentisate 1,2-dioxygenase	CGTGCACTTTGTCGAGCATT	CTAAGGTAGCGCTGGGTAGC
ATEG_06713	gentisate cluster TF	GAGCGGCATTTATTCGCCTG	AGCGGAGCGTTAAGATGACC
ATEG_06714	maleylpyruvate hydrolase like	CCGCAATGGGGGTTCTCTAA	CGACACACTCTCGTCCTGTC
ATEG_07116-17	gentisate 1,2-dioxygenase	GCTGTTCAAGTCGTGGCCTA	TCAAGCCTCGCCTGCATATC
ATEG_07652	aromatic monooxygenase	GCGGACTCCATGTCCATGTC	GACTGGCGCACCTGATAGAC
ATEG_08614	aromatic monooxygenase	CCCTTTTCGAAGGCTGGGAT	GGACGGCGTCTCCTAACATT
ATEG_08615b	phenol monooxygenase	TCGACAAGTCTATGGCGACG	TCGCAAAAATGCCTTGCGAC
ATEG_08616	nicotinate dehydrogenase	AGTGAAACGCATGGGAGGAG	CTGCCCAGTGGTCATCATGT
ATEG_08618b	nicotinate cluster TF	CTCATCCACCAGGCGACTTT	GCAAAAGAGCTGGTTTCGCA
ATEG_08898	aromatic monooxygenase	TACTGCGACAAAAGCCACCA	GCCGCAGAGCCATCTGAATA
ATEG_09120	aromatic monooxygenase	GGGCATCGTATTCAGCCAGA	ACCCAATGCGTTCGTGGATA
ATEG_09602	catechol 1,2-dioxygenase	CAGGAGGAGTTCAACCTGCG	GGGCGGAAGGGATGTCTATC
ATEG_10004	Baeyer-Villiger monooxygenase (BVMO)	GTGCAGTACCAGCAAGAGGT	ACACCAGCGTCGTATAGCTG
ATEG_10009	2-aminomuconate-6-semialdehyde dehydrogenase (AMSDH)	CCAGAAGAGCATCTGGTCCG	CCGCGAGAGTCAAGTACGAT
ATEG_10009-10	3-hydroxyanthranilate 3,4-dioxygenase (3HAO)	TTCCCATCAAAGAGGGCGAG	CCTTGGTGCAATACCACCGT
ATEG_10010	aminocarboxymuconate-semialdehyde decarboxylase (ACMSD)	GTGTCACGCCGATGGAACAT	GGGAACGGATAGTCACTGCC
ATEG_10011	lower kynurenine cluster TF	ATAGATCGGACGGGACACCT	CGAGCCACTATGATAGCGCA
ATEG_10014	aromatic monooxygenase, putative	ACTCGCCCTACCATGTCTCT	CTTGCCAGTGTGAATGACGC
