# Supplementary material for: Twists and Turns in the Salicylate Catabolism of Aspergillus terreus, Revealing New Roles of the 3-Hydroxyanthranilate Pathway
Source: mSystems. 2021 Jan 26;6(1):e00230-20. doi: 10.1128/mSystems.00230-20 (PMC7842363; doi:10.1128/mSystems.00230-20)
Supplement: TABLE S2 [file mSystems.00230-20-st002.pdf]

Table S2. Gene sequences of predicted new genes or of poorly annotated genes.

Gene name	Description	Notes	Sequence
ATEG_00348-49	gene similar to *qut* H	New gene predicted in intergenic region between genes ATEG_00348 and ATEG_00349	ATGCCCCTCCGAGTTGTGGTTATCGGCGCCGGCCTCATCGGCCCTCGTCATGCGCAATCCGTGCGCGCGAACCCAGACACCGAGCTGGTCGCCCTGATCG<br>ACCCGTTCCCCAGCGCCGCCGCCACAGCCCAAAAGCTCGACACGAGCTACTATCCCACGGTCGAGGCCATGCTGCAGGCGATGCCGGCACCAGACGCCG<br>CCATCGTCTGCACGCCCAACCACACGCACGTCCCGATCAGCAAGGAGCTGCTGACAGCCGGCGTGCACGTCCTGGTCGAGAAGCCCATCAGCGACAGCC<br>TGGAAACGGGCCGGAGTCTCCTCGCCCTCGCCGCGCACCAGCACAGCACGCACCCCAACAAACCCACGCTCCTAGTCGGCCACCACCGCCGCTTCAACCC<br>GTACCTCCTCAAGACGAAAGAGATCGTCGACGCCGGCCGCCTGGGCCGCATCATCGCCATCAACGGCCTGTGGACGCTGCACAAACCCGACGCCTACTT<br>CTCGCCGCCGGGCGACTGGCGGCGCGCCCGCGCCTCGGGCGGCGTCTTCGCCATCAACCTCGTCCACGACATCGACCTGCTGCACTACCTGTTCGGGCC<br>GATCGTGCGCATCCACGCCGAGCGCACGACGCCGCAGCGCGCCAACCCGCCGCACGAGGCCGAGGAGGGCGCCGCGCTGACCCTGCGCTTTGCGTCCG<br>GCGTCGTCGGCACGTTCCTCGTCTGCGACGCCACGCCCTCCCCGCACAGCTTCGAGGCCGGCACCGGCGAGAACCCCATGATCCCCGCGGTGGGCGCCG<br>CGGGCGACTTTTACCGCATCTTCGGGTCGGATGCGTCGCTGAGCGTGCCGGATATGACGCGCTGGAGCTACGATGGGCGGCCCGAGAAGAGCTGGACG<br>CAGCCGCTCACGGTGGAGAGGTTTGAGGTGGTCGATGCGACGCCGTTTGATCTGCAGCTGGCGCATTTTGTGGATGTCGTGCGCGGCCGCGCGGCGCC<br>CCGCTGTTCGGGCGAGGACGGGCTGCGCGCGCTGGTGGTGTGTCAGGCGGCGCGGAAGGCGCTGGAGACGGGCACGACGGTGGATTTGGATGCGGA<br>TCTGGAGGCGAGTCTTTGA
ATEG_00350new	quinate 5-dehydrogenase	New gene prediction of the poorly annotated gene ATEG_00350	ATGGCTGCTCCTCCCACCACATCCGCCTCCTCCGGCTCACCCCCCGAAGAGCCCATCCTCATCCCCGACCATCTCGACGGCGTCGCCTACCTGTACGGCCA<br>CCCCCTGCTGAACTCTCTCTCGCCGCCCCTTCACCAGACCGTCTACAATGCCCTCGGTCTCAACTGGACGCAGATCCCGCTGTCCAGCGTCTGCGGCACCT<br>CCGCCACCTACCCGCCGCCCTACACGCGCTCGCCGCCCATCGACAAGTTCCTGGCCTCCATCCGCGCTAACCCCAAGTTCGTCGGCTCGTCCGTGACCATG<br>CCGTGGAAGGTCGCCATTATGCCGCACCTCGACGACCTGACCGAGGACGCCCGCCAGGCTGGCGCCTGCAACACCATCTTCCGCGACCCCGCCGGCCGC<br>TATGTCGGCACCAATACCGACTGCATCGGCATCCGCGAGGCCCTGCTGCAAAACTCCCCCAACCCGGCCGCCTTCCGCGGCCGCCCTGCCCTCATCGTCG<br>GCGGCGGCGGCACCGCCCGCACCGCCATCTACGTCCTGCGCAAGTGGCTCGGCGCCAGCCGCATCTACATCGTCAACCGCGACCGCGCCGAGAACGCCC<br>GAAGGAGGTCCGCGCCCGCGACGTCCTGCGGACTTTTCTTGGGGTGCGAGCCGGCGCGGCGGAGCCCCCAGGGCGTCATTCTGGAAATGTGCTACCAC<br>CCCGTGCCCTGGACGGAAATCGCCGACATGGCGTCCTCCGCCGGCTGGAAGGTCATCCTGGGCTCCGAGGCCCTCATCTGGCAGGGTCTGGAGCAGGC<br>GCGGCTCTGGACCGGCCAGGACGTGGTCGCCGTGCCGGGCGTGGTCGACCGCGTCAAGGAGGTTGTCAACAACGCCATTGCCGAGCGGTCCCAGACCA<br>AGCCGAAGATGTAA
ATEG_00351new	gene similar to *qutG*	New gene prediction of the poorly annotated gene ATEG_00351	ATGGCAGTCCCCGAACTCACCCAGCAACAGCTGGACGAGATCTACGCCTTCGCCGTCGATCTCGGCCGCAAGGCCGGCCAGTTACTGCTCGAGAGCATC<br>GAGAAGCGCATGGACGGCGAGCAGAGCCGCGAGGCCGACGAGAAGGAGAATGCCGTTGATATTGTTACGCAGACAGATGAGGACGTGGAAACGTTCA<br>TCAAAACCGCCATCCAACGCCAATACCCCTCGCACAAATTCCTCGGCGAAGAAACCTACGCCAAAGGCCAGTCGCGCGACTACCTGATCGACGAGAATCC<br>CACCTGGTGCATCGACCCGTTAGACGGAACAGTCAACTTCACCCACATCTTCCCCATGTTCTGCGTCTCCATCGGCTTCCTCGTCAACCACCGCCCCGTGAT<br>CGGGGACCAGCTGTTCTCCAGCTGCGTCAACCGCGGCGCCTGGCTAAACGAGACACGGAGGCTGCCGCTGCTGCGCAACCCTGTGCCGCCGATGCCCG<br>CCACCGCGCCCAGCAAGTGCATCTTCGCGTGCGAGTGGGGTAAGGATCGTCGCGATATCCCCGACGGCAACATGCACCGCAAGATCGAGAGCTTTGTTA<br>ATCTCGCCGCGGAGGTAGGCGGCCGCGGTGGCAAGGGCGGTATGGTGCATGGGGTGCGGAGTCTGGGCAGTGCGACGCTGGACCTGGCGTACACGG<br>CTATGGGGTCGTTTGATATCTGGTGGGAGGGCGGTTGTTGGGAGTGGGACGTGGCGGCGGGCATTGCCATTCTGCTGGAGGCTGGTGGGCTAGTGAC<br>GACCGCCAACCCACCCGAGAATCCAGACACGGCTCCCATTGAGGATGTGCGGCTGGGTAGTAGGTTGTACCTGGCGATTCGTCCGGCGGGCCCGTCGG<br>CGACCGAAACCGGGCGACAGTCGCAGGAGCGCACGGTGCGCGAGGTCTGGCGCCGGGTGCGCCAGCTGGAGTATACGCGGCCGGGGGCATAG

ATEG_07116-17	gentisate 1,2-dioxygenase	New gene predicted in intergenic region between genes ATEG_07116 and ATEG_07117	ATGCCTTCAATCGCCGAACTCTCCTCTCCAGCCGTAGCCCAAAAGCTCGACTCCCTCTCCACACGCGCCACAGACAAACCCACCCTCGAAGCCGAAATCAA<br>AACCCAGTCCCTAATCCCCCTCTGGAACACCGGCGCGCCCATCTCCGCGCCCACCCCACGCAGCAAGCAAATCCCCACAGTCTGGCACTACGCCGACACA<br>AAGAGCCTGCTCCTCCAAGCCTCCAAGCTCGTCGACGCGCGCGAGGCCGAGCGCCGCGCCGTGCTGATGATCAACCCGGGCCGCAACGAGTCGCCCTTC<br>ACGCTGGACACGCTCCTCGCCGCGCACCAGCTGATCCTGCCCGGCGAAAAGGCCGTCTGCCACCGGCATACGCCCTTCGCCGTGCGCTTCCTCATCGAGG<br>GCGCGCGCGGGTACACCGCCATCGCGGGGCGCAAGATGTACATGGCGCCGGGGGATCTGATCATTACGCCCGTGTGGAACTGGCACGACCACGGCAAC<br>GACGGCAACGAGAACGTCATCTGGCTGGATGGGCTGAATATCCCGCTGTTCAAGTCGTGGCCTATTGACTTTACGGAGCATTATGGCGAGGAGTTTGGG<br>CGCGATACGCATGACAGCGAGGTGTTTCCGGATGATGAGGAGCGCGTTGCGCAGATGAAGTTTTCCTGGGGGGATATGCAGGCGAGGCTTGATGCTGA<br>GCCCGGCGAGGCGGCCGTCTGTGAGTATCTGCTGCCGGGGTCGGGGCGCAGTGTGTCGACGACTATCGGCGCGTATGCGGAGCGTGTCAACGCCGGC<br>AAGGTGTCCGCGCGTCGCAAGGAGACTACCAACCATGTCTTTCAGGTGCATCGGGGGTCTGGGTGGACGGAGGTGGTGAATCTGGATGGATCGAGGA<br>ACACGCTGCGTTGGGGACCGGGAGATGCCTTTGCGATTCCCAGCTGGCATGAGTTCCGCAATTTTGCGGACGCTGATGAGGCTGTGTATCTGTTTTTCTT<br>CTCGGACAAGCCGATGTTGACGAATCTGGGGTTCTGGCGAGAGGGTTGA
ATEG_07908-09	3-carboxymuconolactone decarboxylase	New gene predicted in intergenic region between genes ATEG_07908 and ATEG_07909	ATGCGTCTGCCGTACGCCCCCAACACCCCTCCGACCGCCGACCAGGACACGGCCGACATCTACCAGCGCATTGCCGAGCGTCGCCATCCCCGCCCTCTGA<br>TCCCGCTGGATCTGTCGCTGCTGCACTCGCCGCCGGTGGCCGATGGCTGGAACAGCTTCCTGGGGGCCATTCGCACGCGCACTGTCGTGGACCAGGGCC<br>TACTGGAGCTGGCTGTGTGTCGTGTGGCCGTCTTGACCCAGGCCGTCTACGAGTGGAATGCCCATGCCCCGCTGGCATTGAAGGGCGGCATCAGCCCGG<br>AGTCGTTGCAGGCGGTGCGTACGCTGCCTTCGACTGCTGCGGGTGAGGATCTGGCTGCGACGAAAGAGGCGCTCGCGAAGAGTGCCCTGTCTAGCCGT<br>GAGCAGGCGGTCGTCCAGTATGCCGATGAGATGACGCAGACGGTGAAGGTGCAGGATGCGACTTTTGCCCGGCTGCAGACGGAGGGCCTTTCGGACA<br>GGGAGATTGTCGAGTTGACGACGGGCATTGCGGGTTACAACTGCGTCAGCCGGGTGCTGGTCGCGCTGGATGTGGGAGAGAACAACGATAAGCAGAT<br>GAAGTCGGTGGACGAGCTGGTGCAGGCTTTGAAATGA
ATEG_08211-12	4-amino-4-deoxychorismate lyase	New gene predicted in intergenic region between genes ATEG_08211 and ATEG_08212	ATGGTTTCCGACTTTCAGATCATCTCCTCCCTGCGCTACGATCCATCTCTCCCAGCGACCGTCCAACACTACGCCGCAGACTCCTATCCCGACCCCCTCCGC<br>TCCGCCTACTACCTCCTCCCTTATCATCTAGATCGGCTACGCGACGCAGCTCAATGCTTCCAAGCGCATGAGTTTCTCCAACAGGATCTGACACAATTCGTC<br>CGATTCCTCGACACCTTCATCTCGGATCCGACGAAACCATGGCGTCTGCGCATCGTCCTCGGTCGCAACGGATCCTGTACCGTTGAACCAAGCCCAGCGG<br>CGCCCATGGAGACCAAGAACCTCCTCCTCCCTTGCAGAGCGCCATCCGATCCGAGCGGATGGCGAGTCTATATCGATTCGCAGACCACGACCCCCTCCGC<br>ATTCACCACACACAAGACCACCGCGCGGGACGACTACACAGCGGCGCGACTCCGGTCCGGCATCCTCTCCCCTCAGGACCAAGCGGAAGTGCTTGTGGT<br>GAACCCCCAGGGTGAAATCATGGAGGGAAGTATCACAACTCCGTACTTTCGACGACGGACACAGGGGTCGGCGGAGGCACACGGAGATCCTGGTCCG<br>GAGTGGATCACGCCACTGCTCTCCTGCGGCGGCAACTCGGGGACTACGAGACGATACGCAATGGTGCATGGGTTCTGTACGGAGGGAGTCATCCTTGCT<br>GCAGACCTGGTCGATGGAGAAGAATGTTGGCTCAGTAACGGAGTCCGAGGCTTTATGCGCGGTCGAGTCGTGCGCCGTTGA
ATEG_08219a	RmlC-like cupin domain dioxygenase	New gene prediction of the poorly annotated gene ATEG_08219	ATGGCCTTGTGGAACAAACAGCCCCCTCCATCGAGGGTCCCATATGCAATCCCGCAGCTAGAAGGCGAGCGCCTCACGATCCCAGGGAGCAAGGGCGT<br>GTTTCGCATTCTGGCATCCTCAAAACAGACGAATGGCCTGATGGCCGTCTTCCAGAGCGGCGCAACGCTATCTGACGCGCCCGGATTCCACTACCACAAC<br>CAGGCGCATGATGTCTTCCTCGTAACAAAGGGCTATCTAAAGCTCTGGAACGGGGACAAATGTCGCATCATGGGACCGGGTGATTTTGCCTACATTCCTC<br>CGAAAGTCATCCACAACCCCGAAATGCTAGGCCCCCACACGGAGACCTACGGCGTGGTCACCCCCGGCGACTGGATCGATTTCTTCCGCTACGTCGCCGA<br>GCCCTACGAGGGCCTCATCGTCCCGGAGGACGACAACCGCGACCTGAAGGCGCTGCTGATTCCGAAGGTCATGGCCGCCAAGGGCCAGTTCGACGTCG<br>TCTTCCAGCCGGACTACCAGCCCCCGGAGGTGGGCGAGTGGACCGCCGACGATGAGACGCTCCCGGAGTCGTCGCAGCCGTTCTTTCTGAGGGCGAAT<br>ACCGGCCCGCGTTGGATGCTGGGCGGCGTCATGTCGCGCCCGTTCGTTACCACCACGCAGAGCGCGGGCGTGTGCGCCATCTCCAGCATTGAGTCCTCA<br>AATGAGTATGGTGCGTCGTTGTTGTCGAAGTATATGACGTTCCAGAGTGTGGATCATTGTCTGTGCGTGCTGGAGGGGACGTTGGTTGTAAGGTTGAAG<br>GGGCAGTCGGAGAGCGTCTTCCGCGAGGGCGAGACGGTGGTCATTCCGGCGGGACAGGCGTTTGCGCTTGACTTTGGCAGTAAGTATGTGCGGGTCTG<br>GTCGTTTACAGATGGCGATGGCATCGAGACGCTGGTGCATCGGCTTGGCACGCCGTTTGAGGGCGTGGTGTTGCCGGACAAGGAACTGGAGTGGGATT<br>CTACAAGAGTTGGGGTGGTGGCGGCGGAGCTGGACGTCACGATTGAACAGTGA

ATEG_08219b	Zn(2)-C6 fungal-type DNA-binding domain	New gene prediction of the poorly annotated gene ATEG_08219	ATGATGCAGCGTCGGACGCATCCACCCGCATCTGCACGTCCCGCAGCGGATGACGCGACTCCACCGCGCGCGAAACGACCGCGGGCAGCGCGCGCCTG<br>TGACCGCTGTAGACGGAAGAAGTACAAGTGCGACGAGTCATATCCATGTGCGCATTGCAAGAGTGAGTTGCTGGAGGTGTATTGCATGTTACGGAAGC<br>TTACGTGCGCAGAAAGTGGAATCGACTGCGTCTATCAAGGGAACTATCGCGCGCAGGAGAGTAACCGATCCGCGAGCTACATCCTCGACCTGGAGAAA<br>AAGGTCAACGACCTCACGACTAAACTGCGGATTGCGGAGTCCGAGATCGCAGCCAGACAGTCTCCCAGCGCGCAGGCCGCAGCTTCTCAGCCCGAGGG<br>GCCTGTCGCACGGCCCGGCATCGATGCGACACCGTTATCCATGCCACAGGATAGCTCAACACCCCGCGACGAGATCTCACTGCCGTACGACGCCCCCGAC<br>GACGCGGGCGAGATCGTTCAGGAGGAAATCAGCGAGCTGAACCAGCATACTAACGGCATCGAGTTCCACGGGAGCACGTCATCTGCAGCGCTGCTCGG<br>CCATCTCCAGAAGGCGCGCGAGCAGCGCAAACCTGAGGATTGGCTCTCCCATCCCAACGAGCCGCGGTACTCGTTGATCTCGACGCTGCATAACTCGAG<br>CTTTTCGCCGTCGTGCACGACGGCGCCCGTGCGGCCGGTGCCGCTGCAGGAAAACAACTACTACTTTGAACAGGCGCACACGTTCATGAATGGATATTTC<br>GAGAATATCCACTTCGTTCACCCGTTAATCGATAAAGAGGACTTTTATCTGCGCGCGCATGAGCTGTGGTTCGCGCGGGACTGTCAACCGGACCCGAGCT<br>TTGTTGCACTGTATCTCAGCGTGCTCTCGTTTGGCGCGCTGGTTCGCGTGTGGGACGAGGCGACTATTGGGGGCCTCGGGAGGTTCGAGTGGAGTCGG<br>AAGCTGTTTTCCGAGGCGCAGTTGTATCTGAACTACCTGCAGTTCTCGAATAACCTGGACACGGTCCAGTGCTTGTATCTAATGAACGAGCTCAATCCGA<br>ACTTGGCGTATATGTACCTTGGTCTCGCGGTGCGCACTTGTCTCGCGGCGGGCTTCAACCGGGATGTCCGCAACTGTACCGATAGTCGGTCGGGATGGA<br>TTTCAAAGACATGGTGGGGACTATTCTCTCTCGAAATGTATGTTTGCCTCCATTCGTACTGCGTTGTTGCTGACGACACAAGTGAAATGAGCTTCTCGGTG<br>GGCCGTCCCGACACTCTAGGCATGGACGAGTATCACAACCGTCCACTTCCCGAGCGGGACGACTCCGAATACGCCATTATCCCGTGGATGGTCGACTTCG<br>CTCAAATGACCCGTAAGGTGTCAGTACAAATATACCATTCACAGATTACATTACAGGAAAAGCTTCAAGTTGCACTTCAGGTCGAGGCAGAGCTGGACCA<br>ATGGATGCTCAAACTCCCAGACAGGATAAAACCAGACATCATCCGACATGATGTTTCTCCCAGGGCGCTACGAGATCCGAAATGGGCCAGACGACAGAA<br>GCTGGTCCTTGCCATTCGCTACTACAATGTGAAGATGCTTCTATTCCGCCCGTTCCTCGGCCACTTCACCCGAAAACTACGCCATACGCCGAGTGAGCTGG<br>AAGAGACGATTGCAAAATGTCTCGATGCTGCGATGAGGACGATCGAGGTCATTTACGACATCTACCGCGTCCATACGTTTTTCCGATGCTGGTATCTCGG<br>CATATGCCCCGACACACTCCCGCTGCGGCGTTCAGTCGAAATGGCGGTGGAGATCCTGGAGGCAATGGACGAGTCCGTCGTGGCACGCAAGTCGGTCG<br>AGATCATCAAGCACTACCTGAACGAATTCCAGGCCTCGGAGGCGCAGACAGCCGTGGGAGGCGACAGTACCGAGGGGAACGCTGCAGCGTATGTCTCG<br>GAGACGGCCCCCAGTCTCGGGGGGTTTGACGTGCCGGAGTGGGCCTACGGGTTTGGATTCCCTGAATTTTCTTTTGAAGGGATCGCGCGGCTGTTCGAC<br>GACCTAGGCGGATTGCCTATGTTGGACAACTGA
ATEG_08615a	short-chain dehydrogenase/reductase	New gene prediction of the poorly annotated gene ATEG_08615	ATGTCTAGAGTAGGTCGGACTGCGTTAGTCACCGGTGGTGCTCGAGGCTGCGGTCTTGCATTTGCTCGTGGACTCGCTGAAGCCGGTGCTAATGTGGCT<br>ATATTCGACGTAATCGATCCGGAGGAGGGTTTCTACGTTATACAGAAAGAATGCGCTGTGCGGACAGCGTTCTACAGGGTCGATGTGGCCTCCAAAGAA<br>TCCCTCGCCAATGGCTTCCAACAATTCCAAGCTGATTTCGACAATGCACTCGACATCTGCGTTCCCTGCGCGGGCATCAACCGCCACCTTCCCTTCCTCGAG<br>TTCACCTACGAAGAGCACCAGAACCTATTGTCGGTCAACGTGATGGGTCTATACTTTACCGCTCAACTAGCCGCAAAACAGATGATTGCCAATGGGACGA<br>AACATGGTAGCATCGTGTTGGTGGCCAGCATGGCCAGCCATATCGCCGTTCGCGATCAGCTGTGCAGCGCATATTGCGGGTCGAAAGGAGCTGTCCGCG<br>CCATGTGCCCAGCCATCGCGAAGGAGCTTACACCATATGGCATCCGAGTGAATACGATTTCGCCGGGATATGTCCGCACGGAGATGACTGCAGCGGTAT<br>GTTTCGATCATTTGAGAGCAGATCCACACACTGACAAATATCAGTTCCCCCATCTGTTGGAAAAGTGGAAAAGCGAGGCGATCAGTGGGCGAGTGGGTG<br>AGCCGGAGGATATCATGGGGGCTTGTGTGTTCCTGGCCAGCGACGCCAGCGCCTTCATGACGGGACAGGATGTGGTCGTGGATGGGGGTGTGACGCG<br>ATGGTGA

ATEG_08615b	phenol monooxygenase	New gene prediction of the poorly annotated gene ATEG_08615	ATGAAGCGCACGTTATCAGAGGCTAGACTGGAGCGTAGCACAATGGCTCCTAGTGCAGACCACGAAGGATTCAGCGATAGCCCTGTCGAGGCTACGAC<br>AACGGTCGTGGTTGTGGGCGCCGGGCCATCTGGATTGATGCTTGCCTGCAATCTAGTCCGGTATGGAATCGATGTGGTTATTCTCGACGATCGTCCTGAC<br>AAGACATCCACCGGCAAGGCAGACGGCATGCAGCCCAAGACGATCGAAACCTTCAAACAGCTTCGCCTAGCCGACCCATTGTTGAAAAATGCCGCCCGC<br>GTGTACGACATCGCCTTCTGGGAGTCCACGGCAGACACACCGATGCACCGCACTGGTCGTCAGATCCACTACCCAGCACATCTTGTCGGCGCCTCCGACC<br>CCTATATTCTTCTTGCGCACCAAGGCATGCTTGAGGACATTCTAATTCAGGACATCGAAGCCCGAGGCGTGTCAATCCAACGCAATAGCCCGTTCGTGTC<br>GTGTTCGAGACTACCAGACAGTAGTCAACTGCACGTCTCCTATCTAGACCTGACGACCAAGACCGTCAAGAGCATCCGCACAGACTATCTTGTTGGTTGC<br>GATGGCGCCCGGTCCAAAGTGCGCAACTTCATACCCAATGCACCCCTCGAAGGGGAAATGACCAATGCATCATGGGGCGTATTGGACGGCGTAATCGA<br>GACCGACTTTCCCGACCTCTGGAGCAAAGTCGCCGTGCGCTCACACTCAGCCGGCTCGATCCTCTGGATCCCGCGGGAACGAAACATGACGCGCCTGTAT<br>GTTGAGCTGAGTTCGACCGACGGCGAGCGGGTCGACAAGTCTATGGCGACGCCGGAATACGTGATGCGACGGGCGAAGGAGGCCATGCACCCGTTCC<br>AGCTGGAATGGAAGTCTATCGAGTGGTTTGGGAACTACGTCGTCGGCCAGCGAGTCGCAAGGCATTTTTGCGATCCTTATCTGCAGATATTTATTGCTGG<br>TGATGCGGGCCATTGTCACTCCGCCCTCGCAGCTCAAGGTGCCAACACGAGTATGCACGATAGCTTCAATCTCGCGTGGAAGCTGAACCTCGTCTCCCGT<br>GGACTGGCGCAGAAGTCTCTCCTTGCAACGTATGAGGACGAGCGTCGCAAGATTGCCTACGATCTGATCAATTTCGATGCTGAACATTGCAAGGCATTTG<br>CGCAGGGAGAGGCCGCGCTGGCGAAGAACTTCGACGATAACATCCGTTTCATCTCTGGGGTTGGAGCGGAGTACTCCAAGGGCGTCCTCACTCGTATTG<br>CCCCCGAGAAGAACTATCCTCTTCCTGCACGCCCTGGAGCGTTGATGATCCCGTCGAAAGTCACACGGTATATCGATGCCAACCCTGTCGATATCCAATTG<br>GATATTCCTATGCTGGGCCAGTTCCGAATCTACCTGTTCGCACCTGATGTCGTCACTTCTATGCCGTTCCTAGAGGATATTTGTGTAGGATTAAGTAAGGG<br>GGACTTTCTGAACCCGGTGTCGTCCAAAGCTGCCCAGTCTTACAAGGAGCAGCAACGGAAACAGGCAGAGGCAGATGCATACATCACACCCGAACGCTA<br>TACTGCTGTCTCTGACATCTTCACGTACGCTTTGGTGACACAGTCTCCCAAGGAGGCGTTTGAGATTGCCTGCCTGCCGTCTGACCTGCAGAAGAGTCGA<br>TGGACTCTGTATCTGGACGATGTTGAAGGAGAAACATGTACAGCCAAATGGATGGGACCATTGGCAAAGACGCACACAGGCATTGTCGTCGTACGACCA<br>GACGGGTATGTCGGAGCGATGGAAACCTGGACAACTGATCAGGGCCGTCTGGCAGTGCAGTGGATCGAAGAATACTTTAGAGGCTTTCTTTGA
ATEG_08618a	MFS transporter superfamily	New gene prediction of the poorly annotated gene ATEG_08618	ATGATTACTATGGAGAAACGAGAAATCACGGGAGACCGCATGTCGCTAGATCCAGAGGCCAAATCAGGGCAGTCGAATAGCAATCTTGCCAATGCGAA<br>GACAGATGGTCTGGATAAAGACATTCATCTCGAAGGAAATGACTACTCCTTGCTCGTTTTGTTGTTCTATATCCCATTCGGGTTGTTTGACCTCCCGTGGA<br>ACCTGCTCATCAAGCGGTTCTCTGGCCGGTTGATGCTCTCTTTGATGTTCGAAGCCGGCTTCTTCTGTGGAGCGACGTTCTATTTCACTTTATTCTACACCC<br>GTGGTGAAATGGGCTTCCGCTTGGCCATCATGCAGTCCTTTGCTGTGCTAGCTTCTGCATTCAGCGGACTGATCTCCTTTGGCGTATTCCAGATCGGCTAT<br>CCCGCAGTCCACGGTTGGCAGTGGCTGTTCATCATTGAAGGGGCAATGACCCTCATCACCGGAATCGTCAGCTTTTGGTGGCTGCCGGACAACGCTCAA<br>ACGGCCTGGTTCCTGAACGACCGAGAACGAGCTGCTGCGACAGCCCGACTATTGCGTGACACTTCATCCGAGGTAGAAACTGAACTCGATCTGAAGTCG<br>TGTTTTCGGACATGGAAAGACTGGAAGTTCCCCATTTGGTGTATCATCACGTTCACATACCCGGTGGCCTACGCCACAGCGATGAACTTCTTCCCGCTGAT<br>CGTACAGCGCCTGGGATACTCCGTCATCAAGACAAATCTATGGACGGTGGCGCCCAATCTCGTCGGCGCAGTCGTACTGCTGTGTGTTGCAAAGTCATCA<br>GATTATTTCCGCGAACGTACGTTCCATATAGTCTTCTCATTGACACTCTCACTGGTGGGCATGTTGGTGCTGTCCACCATTGACGTCCTGGAAAACAAAGG<br>CGTCTCATATTTTGCATGCTTCCTCATGGCTGCTGGGGCATACATCCCATCATGCCTAGTCCACGCGTGGCACAACAACAACAATGTTCATGAGAACTCAC<br>GAGCCGCCAACACGGGGTTCTTTGTGGGACTTGGGAATATTGCGGGGGTTCTGAGTGCCGCCACGTTTCGTACTGAATATGCTCCGAAGTATGCATGCC<br>CCTCATTCTGCTGCTGGTCCTTGCGTTTGCTGATGCTTGTAAGGTATATACCCAGGCTGGTAGCTACCTGCTGTTGCAACGGAGTGTGTATCGTCTTTGCA<br>ACGGCTCTAGGACTGTGGATGCGCTTTGAGAACAGACGACGGGACAGGGAGCAGGGGGTCAAGCTGCGGGCAGGGGCCGTTCCTACTAGCGACCTGG<br>TGGATGGAGAAGAGTCTCCTGAATGGCGTTATTTCCTGTAG

ATEG_08618b	zinc finger C2H2 superfamily	New gene prediction of the poorly annotated gene ATEG_08618	ATGAAGAACCACGTCTGCCAGTGGCCTAGCTGTGGAAAGAGCTTTACACGCGCGGAGCACTTGCGCCGCCACGCGTTGAATCATGAACAGGCGCAAAAT<br>GGCTATACTTCAAGACACATGATGCGACATGCCAAACGCGACGAAGAGGCAGGAGGACCGGGCTTGGGAGTGCTGGAGACCCGTAAACGCACTCGTCG<br>TGCGGGTGATGGGACGATTATCACTCGGCCTCCAAAACGGCAGTCTCGCCCTGCACACTCACAGTCCACTACCTCATCATCCATCTCTATAGCGAGCTCGC<br>GAGACCAAAATTACGGTGAATTAGATGCCCATACGCCGCATGGGGCACCTGTATCGCCTCCTGGATCTGCCAGCGATCCGCCATCGCTGTCTTTGGATGA<br>TACGGATGCCCTGCTGGCACCGATGATGCCCGGGGGCCCATTTGAGCCCTATGTCGAACCCATCCCGGGTCAGTTCGATGCAGCAGATGGTTCATTCAG<br>CCTGGGGCTAGACACAACAGCCGACTTTTTCAACATTGATACGGCCACCGATTTCAATATGCCCTTTGCTGCAACCCATAACTACAACTGGCTGTTCGACG<br>TCTCATCGCTCGACGATGCATTTGATCAGTTCGACGTGCCTCTCGGTTCTGATATGATGACGTTTGCCGACCCTGTCGATCCCTATCGAAACAACACTGCG<br>CTTGAGTTTCCTGAGATGAGAGCTTTGTTCGATCAGGATGGCTCATCAGTCCTGCTACAGGCAGCCTCGTACGTTGAGCGAGGGACTCTCGTAGAAACCG<br>CGCAACCCATTGGCATGCCTGATGTGGTCGATATGGAGTGGATGACAGGGTCTTCTGTCTTCGAGACGAGTACGCCTCCCCATCTCCCTCGTCTGAATGA<br>AGAAGCCCGGCGTGGCATTCTCACACTTGTGGCCCAAACATCACCGGTTGGAATTGATGGCAGTGCCACAGCTCTTGACTCACCTTTACTTTCTATGCAAT<br>CTCTGCAAAGCTACTGTGATCTGTTTTTCACTAGATTCAACATTACATATCCTCTCATCCACCAGGCGACTTTCAATCCCAATGCAATTGAGCCGGTCTTTCT<br>CGCGGCTATCCTGTCGATGGGCGCCACCTACAGCAGCCGCGAGGCGCATCAGCTGGCGGTGGGCATCCATGACGCTCTGCGAAACCAGCTCTTTTGCCA<br>TGCAGACTTCTCTCCCCAGCCCGACCTCTGGGTGTTGCAGGCGATGCTGCTTATCGACTGCTTCGGGAAGATGCGGGCTGGCCCCAAGCAGCGCGAACG<br>TGCGCAGCTTTTCCACTGCGTGCTGATAAAACTCATTCGGCGCAGTAATTGCTGTACCATCCGAACCCAGGGCTTGTCGAACCGACCGAATGACCTGGAT<br>TTCGCTTGGCGACAGGCGATGGAACAGGAGCAGCGCAAGCGACTGGCGATGCATTGTTTCATGTGGGACACACAGCACGCGGTTTTGTTCTCGCAGTCC<br>TTGTGCATGTCGGCGTTTGAGATACGATCGTCTCTACCCTGTAGTGCGGCCGCCTGGGAAGCGAACACGGCAGACGACTGGGCTCGTCATGCATCTCGT<br>GAACAGGATCATGTCTTCCTCAATGTCCTGAAGGGTTACATTACCCCAGGAGCCGTGTCCCGCCCACGGGACCTCAACTCCCTTGCACGCATTGTCGTGCT<br>ACATGGACTGATGTCTGTCTCGGCTGATTTGAAAAGGCGAGACCAGACGACCCTGCGGTCGGAGACACCGGAGAAAGTGGGTGCATGGACGCCTCGAA<br>TGGGCCGGTCTTACGACTTGTGGAAAGTCGACTTTGATGCCGACTGTCTGGCAATGAAGCTCGGCCAAACCGCGGATCCCCGTCGCTTCACGGGAGTCA<br>AGACAGCCACGCATGCCCTCTATCGAGCAGCTCATCTAGCGCTCAACGTGGAAGTTTTGGATTTGCAGATCGCAGCTGGAGCTACACAGATTCTTGGCCG<br>CACAGTGACCGATGACGACCGAGAACGTTCACGGCGACATCTTCCCAGGTGGCTCCATGGGGAATCGGGTGCTTCACTTGTAGCTGCGCGCCATGCGGC<br>GTGCCTCCTACATGATGCCGTCTTGAGTTTGCATGATTGGGAGCAAACGGACGCCTTCCATTTCCCGTGGTGTCTCTATCTGGCGGCACTGACATGCTGG<br>GCTTTTCATCGAGGCATGGACGATCATCTTTCACAGGAACGAATTGGGACTGACATCTCTTCATTGATCGTGGCGATGACGACATGTCCGAGCATGCCGG<br>AGTTGGCTGCTCTGGGAGGGAAATACGACACAAGGGGACTGTTGATGGTGATGGCGCAGCAGCTGGCGACCGTGCGATGGGCAGTGGTTCATGATGC<br>TATGAAGGTGCTGTTGAATCTGTCCAAGTAG
ATEG_10009-10	3HAO, 3-hydroxyanthranilate 3,4-dioxygenase	New gene predicted in intergenic region between genes ATEG_10009 and ATEG_10010	ATGATTCCTCCCTTTTCCTTCCAGAGCTGGTTGGCGGAAAACCAGGACAAGCTTTGTCCGCCCGTCAACAACTACTGTCTGTACGACGGCGACGACTTTAC<br>GCTGATGGTGGTGGGAGGGCCAAATGAGCGCAATGACTATCATATCAACCAAACGGAAGAGTGGTTCTATCAGGTCAAGGGGGACATGCTCCTCCGGG<br>TTGTCGACAACGAGACCTTCCGCGACATTCCCATCAAAGAGGGCGAGATGTTTCTGCTACCATCCAATACCCCCCATAACCCCGTGCGATTCGCCGACACC<br>ATCGGGATGGTCATGGAGCGCAAGCGGCCGGAAGGAGTGCTGGACCGACTACGGTGGTATTGCACCAAGGGAGCGCATGAGAAACCCTCGATGATCC<br>GCGAGGAGGTCTTCTACTGTTCGGACTTGGGCACGCAGTTGAAGCCGTTGATTGAACGGTGGCAGCGGGATGAGGAGAGCCGGCGGTGTGGTGCGTG<br>TGGGAACATAGCCGATCCCAAGTAA
