# Supplementary material for: Twists and Turns in the Salicylate Catabolism of Aspergillus terreus, Revealing New Roles of the 3-Hydroxyanthranilate Pathway
Source: mSystems. 2021 Jan 26;6(1):e00230-20. doi: 10.1128/mSystems.00230-20 (PMC7842363; doi:10.1128/mSystems.00230-20)
Supplement: TABLE S3 [file mSystems.00230-20-st003.pdf]

Table S3. Secondary metabolite gene clusters of *Aspergillus nidulans* (blue) and *A. Terreus (orange)*. Boundaries were manually curated from data obtained in the literature and by using antiSMASH and or from JGI MycoCosm.

Name of cluster	Type	Homologous known ge	Core genes	JGI SMURF	antiSMASH	Manual/**experimental***	Reference
AN0016 cluster	NRPS, RiPP	Fungisporin (100% of g	AN0016/AN0021	AN0015 - AN0016	AN10009 - AN0024	AN0015 - AN0025	Inglis et al (2013) BMC Microbiol 13, 91.
AN0043 cluster	Terpene		AN0043	-	AN0039 - AN0046	AN0042 - AN0044	Inglis et al (2013) BMC Microbiol 13, 91.
Monodictyphenone (mdp) cluster	PKS	Emericellin (100% of ge	AN0150	AN0146 - AN10038	AN0144 - AN0156	**AN10021 - AN10023***	Inglis et al (2013) BMC Microbiol 13, 91.
pkdA cluster	PKS	Stipitatic acid (28% of g	AN0523	AN0520 - AN0533	AN0517 - AN11271	AN0523 - AN0533	Inglis et al (2013) BMC Microbiol 13, 91.
Ferricrocin (sidC) cluster	NRPS		AN0607/AN0609	AN0599 - AN0607	AN0599 - AN0617	**AN0607 - AN0609***	Gründlinger et al (2013) Mol microbiol 88, 862-875.
AN0654 cluster	Terpene		AN0654	-	AN0650 - AN11278	AN0653 - AN0660	Inglis et al (2013) BMC Microbiol 13, 91.
AN0840-AN0842 cluster	Betalactone		AN0840/AN0842	-	AN10125-AN0846	AN0840 - AN0842	This study
Asperfuranone (afo) cluster	PKS	Asperfura (100% of ger	AN1034/AN1036	AN1029 - AN1036	AN1026 - AN11289	**AN1029 - AN1036***	Inglis et al (2013) BMC Microbiol 13, 91.
Nidulanin A cluster A	NRPS	Nidulanin A (100% of g	AN1242	AN1242 - AN1245	AN1237 - AN1250	AN1242 - AN1247	Inglis et al (2013) BMC Microbiol 13, 91.
Ent-pimara-8(14),15-diene cluster	Terpene		AN1594	-	-	AN1592 - AN1599	Bromann et al (2012) PloS one 7, e35450.
AN1680 cluster	NRPS-like		AN1680	AN1678 - AN1681	AN1675 - AN1686	AN1678 - AN1681	Inglis et al (2013) BMC Microbiol 13, 91.
AN1784 cluster	PKS		AN1784	AN1780 - AN1787	AN1776 - AN1790	AN1784 - AN1787	Inglis et al (2013) BMC Microbiol 13, 91.
AN1793 cluster	Terpene		AN1793	-	AN1790 - AN1802	AN1792 - AN1796	Inglis et al (2013) BMC Microbiol 13, 91.
pkh cluster	PKS	Asperfura (18% of gene	AN2032/AN2035	AN2031 - AN2043	AN2029 - AN2041	AN2030 - AN2038	Inglis et al (2013) BMC Microbiol 13, 91.
AN2064 cluster	NRPS-like		AN2064	AN11861 - AN2064	AN2057 - AN2068	AN2064	Inglis et al (2013) BMC Microbiol 13, 91.
AN10289 cluster	DMAT		AN10289	AN10289	AN2342 - AN2347	AN10289	Andersen et al (2013) Proc Natl Acad Sci USA 110, E99-107.
AN10297 cluster	NRPS-like		AN10297	AN2396 - AN10297	AN2396 - AN10298	AN2396 - AN11337	Inglis et al (2013) BMC Microbiol 13, 91.
Emericellamide (eas) cluster	PKS, NRPS	Emericellamide A / em	AN2545/AN2547/AI	AN2545 - AN2549	AN2539 - AN2554	**AN2545 - AN2549***	Inglis et al (2013) BMC Microbiol 13, 91.
AN2554 cluster	Cyclic peptides		AN2554	-	AN10325 - AN11348	AN2554	Inglis et al (2013) BMC Microbiol 13, 91.
AN2580 cluster	Terpene		AN2580	-	AN10321 - AN2582	AN2580	This study
Penicillin cluster	NRPS	Penicillin (18% of genes	AN2621	AN2621 - AN2622	AN2616 - AN10330	**AN2621 - AN2623***	Inglis et al (2013) BMC Microbiol 13, 91.
AN2634 cluster	NRPS-like		AN2634	AN2634	-	AN2634	This study
AN2924 cluster	NRPS-like		AN2924	AN2924	AN2918 - AN2928	AN2921 - AN2924	Inglis et al (2013) BMC Microbiol 13, 91.
Aspernidines (pkf) cluster	PKS	Orsellinaldehydes (100	AN3230	AN3220 - AN3237	AN3223 - AN3238	**AN3225 -AN3230***	Yaegashi et al (2013) Org lett 15, 2862-2865.
AN3252 cluster	Terpene		AN3252	-	-	AN3252 - AN3257	Inglis et al (2013) BMC Microbiol 13, 91.
AN3273 cluster	PKS, Terpene	PR toxin (50% of genes	AN3273/AN3277	AN10388 - AN3282	AN3382 - AN10389	AN10388 - AN3287	Inglis et al (2013) BMC Microbiol 13, 91.
Quinoline-5,8-diones (pki) cluster	PKS	Azaphilone (8% of gene	AN3386	AN3380 - AN3386	AN3381 - AN3403	AN3379 - AN3386	Inglis et al (2013) BMC Microbiol 13, 91.
Microperfuranone (mic) cluster	NRPS-like	Microperfura (100% of	AN3396	AN3396	AN3381 - AN3403	AN3394 - AN3396	Inglis et al (2013) BMC Microbiol 13, 91.
Fellutamide B (inp) cluster	NRPS	Fellutamide B (100% of	AN3490/AN3495/AI	AN3487 - AN3498	AN3485 - AN3502	**AN3490 - AN3496***	Yeh et al (2016) ACS chem biol 11, 2275-2284.
AN3612 cluster	PKS		AN3612/AN10430	AN3608 - AN3612	AN10429 - AN3616	AN3605 - AN3612	Inglis et al (2013) BMC Microbiol 13, 91.
AN10486 cluster	NRPS-like		AN10486	AN3911 - AN3917	AN10489 - AN3917	AN3911 - AN10486	Inglis et al (2013) BMC Microbiol 13, 91.
N-acetyltryptophan (ivo) cluster	NRPS	N-acetyltryptophan (1C	AN10576	AN10576 - AN4649	AN4638 - AN4648	**AN10573-AN4640***	Sung et al (2017) Fungal genet biol 101, 1-6.
AN4827 cluster	NRPS-like		AN4827	AN4826 - AN10600	AN9446 - AN4835	AN4826 - AN10600	This study
AN5272-AN5273 cluster	Betalactone		AN5272/AN5273		AN5270-AN5277	AN5272-AN5273	This study
AN5318 cluster	NRPS-like		AN5318	AN5318	AN5312 - AN5324	AN5314 - AN5318	Inglis et al (2013) BMC Microbiol 13, 91.
AN5359 cluster	Terpene		AN5359		AN5356-AN5362	AN5359	This study
AN5475 cluster	PKS		AN5475	AN5475 - AN5478	-	AN5475 - AN5478	This study
Asperthecin (apt) cluster	PKS	Asperthecin (100% of g	AN6000	AN6000 - AN6002	AN5992 - AN6007	**AN6000 - AN6002***	Inglis et al (2013) BMC Microbiol 13, 91.
Triacetylfusarinine C (sidD) cluster	NRPS		AN6236	AN6236 - AN6238	AN6231 - AN6242	**AN6234 - AN6236***	Gründlinger et al (2013) Mol microbiol 88, 862-875.
AN6431 cluster	PKS		AN6431	AN6431 - AN6434	AN10818 - AN6436	AN6431 - AN6437	Inglis et al (2013) BMC Microbiol 13, 91.
Cichorine (pkb) cluster	PKS, NRPS-like	Cichorine (100% of gen	AN6444/AN6448	AN6443 - AN6454 (AN	AN6439 - AN6454	**AN6443 - AN6449***	Sanchez et al (2012) Medchemcomm 3, 997-1002.
xptA cluster	DMAT	Emericellin (100% of ge	AN6784	AN6779 - AN6790	AN6781 - AN6798	AN6784	Andersen et al (2013) Proc Natl Acad Sci U S A 110, E99-107.
AN6791 cluster	PKS		AN6791	AN6779 - AN6790	AN6781 - AN6798	AN6787 - AN6791	Inglis et al (2013) BMC Microbiol 13, 91.
AN11984 cluster	Terpene		AN11984	-	-	AN11984	Bromann et al (2012) PloS one 7, e35450.
AN6961-AN6962 cluster	NRPS		AN6961/AN6962	-	-	AN6961 - AN6962	Andersen et al (2013) Proc Natl Acad Sci U S A 110, E99-107.
AN12065 cluster	PKS		AN12065	-	-	AN12065-AN12066	Nielsen et al (2011) FEMS Microbiol Lett 321, 157-66.
Alternariol (pkg) cluster	PKS	Alternariol (100% of ge	AN7071	AN7061 - AN7074	AN7064 - AN7089	AN7070 - AN7071	Ahuja et al(2012) J Am Chem Soc 134, 8212-21.
AN7084 cluster	PKS		AN7084	AN7081 - AN12290	AN7064 - AN7089	AN7080 - AN7086	Inglis et al (2013) BMC Microbiol 13, 91.
AN7265-AN12400 cluster	RiPP		AN7265/AN12400	-	-	AN7265-AN12400	Nagano et al (2016) Fungal Genet Biol 86, 58-70.
AN7489 (mirC) cluster	PKS		AN7489	AN7485 - AN7489	-	AN7485 - AN7489	This study
Sterigmatocystin (stc) cluster	PKS	Sterigmatocystin (41%	AN7825	AN7806 - AN7824	AN7816 - AN7841	**AN7804 - AN7825***	Brown et al (1996) Proc Natl Acad Sci U S A 93, 1418-22.
AN12331 cluster	PKS		AN12331	AN11024 - AN7839	AN7816 - AN7841	AN11024 - AN7839	Inglis et al (2013) BMC Microbiol 13, 91.
Aspercryptins (atn) cluster	NRPS	Aspercryptins (40% of g	AN7884	AN7873 - AN12002	AN7880 - AN7892	**AN7872 - AN7884***	Henke et al (2016) ACS chem biol 11, 2117-2123.
Derivative of Benzaldehyde (dba) clust	PKS		AN7903	AN7893 - AN7903	-	**AN7896 - AN7903***	Gerke et al (2012) Appl environ microbiol, AEM. 01808-12.

Orsellinic acid/F9775 (ors) cluster	PKS	F9775 (90% of genes sh	AN7909	AN7909 - AN7916	AN7903 - AN7918	**AN7909 - AN7912***	Sanchez et al (2010) Mol Biosystems 6, 587-593.	
xptB-xptC cluster	DMAT		AN12402	AN7999 - AN12402	AN7996 - AN12431	**AN7998 - AN12402***	Sanchez et al (2011) J Am Chem Soc 133, 4010-7.	
AN8105 cluster	NRPS-like		AN8105	AN8105 - AN8113	AN8098 - AN8112	AN8105 - AN8112	Inglis et al (2013) BMC Microbiol 13, 91.	
AN12440 cluster	PKS		AN12440	AN12440 - AN8141	AN8133 - AN8147	AN12440 - AN8144	Inglis et al (2013) BMC Microbiol 13, 91.	
Naphthopyrone YWA1 (wA) cluster	PKS	Naphthopyrone (100%	AN8209	AN8206 - AN8209	AN8203 - AN8213	AN12404 - AN8209	Inglis et al (2013) BMC Microbiol 13, 91.	
AN8376 cluster	RiPP		AN8376			AN8375 - AN8376	Nagano et al (2016) Fungal Genet Biol 86, 58-70.	
Austinol (aus) cluster A	PKS	Austinol/dehydroaustir	AN8383	AN12376 - AN11085	AN8378 - AN8389	**AN8379 - AN8384***	Lo et al (2012) J Am Chem Soc 134, 4709-4720.	
Aspyridone (apd) cluster	Hybrid (PKS-NRPS)	Aspyridone (100% of ge	AN8412	AN8398 - AN8414	AN8405 - AN11609	**AN8408 - AN8415***	Inglis et al (2013) BMC Microbiol 13, 91.	
AN8433 cluster	NRPS		AN8433	-	-	AN8433 - AN8441	This study; Cacho et al (2012) J Am Chem Soc 134, 16781-16790.	
Nidulanin A cluster B	DMAT		AN11080	AN11080	AN11082 - AN8483	AN11082 - AN11080	Inglis et al (2013) BMC Microbiol 13, 91.	
AN8504 cluster	NRPS		AN8504	AN8499 - AN8504	AN8496 - AN8524	AN8495 - AN8504	Inglis et al (2013) BMC Microbiol 13, 91.	
Terrequinone (tdi) cluster	NRPS-DMAT	Terrequi (100% of gene	AN8513/AN8514	AN8506 - AN8525	AN8496 - AN8524	**AN8513 - AN8520***	Inglis et al (2013) BMC Microbiol 13, 91.	
AN8625 cluster	RiPP		AN8625			AN8625	Nagano et al (2016) Fungal Genet Biol 86, 58-70.	
AN8910 cluster	PKS		AN8910	AN8910	AN11143 - AN8915	AN8905 - AN8910	Inglis et al (2013) BMC Microbiol 13, 91.	
AN9005 cluster	PKS		AN9005	AN9002 - AN9013	AN9000 - AN9012	AN9002 - AN9007	Inglis et al (2013) BMC Microbiol 13, 91.	
AN9129-AN9131 cluster	Betalactone		AN9129/AN9131	-	AN9125-AN9133	AN9129-AN9131	This study	
AN9189 cluster	Terpene		AN9189	-	AN9185 - AN9193	AN9189	This study	
AN11191 cluster	PKS		AN11191	AN11191 - AN9214	AN9212 - AN9233	AN11191 - AN9220	Inglis et al (2013) BMC Microbiol 13, 91.	
viridicatin/aspoquinolone cluster	NRPS-DMAT	Penigequinolone (85%	AN9226/AN11194/A	AN9223 - AN9236	AN9212 - AN9233	AN9223 - AN9236	Ishikawa et al (2014) Angew Chem Int Ed 53, 12880-12884.	
AN9243/AN9244 cluster	NRPS		AN9243/AN9244	AN9243 - AN9254	AN9241 - AN9254	AN9243 - AN9245	This study	
Austinol (aus) cluster B	-	Austinol/dehydroaustir	-	AN9243 - AN9254	AN9241 - AN9254	**AN9246 - AN9259***	Lo et al (2012) J Am Chem Soc 134, 4709-4720.	
AN11820 cluster	NRPS-like		AN11820	-	-	AN11820 - AN9294	Inglis et al (2013) BMC Microbiol 13, 91.	
AN9314 cluster	Terpene		AN9314	-	-	AN9313 - AN9314	Inglis et al (2013) BMC Microbiol 13, 91.	
terrein cluster	PKS	Terrein biosynthetic ge	ATEG_00145	ATEG_00133-ATEG_00	ATEG_00138-ATEG_001	**ATEG_00135-ATEG_001**	Guo and Wang (2014) Front Microbiol 5.	
nidulin-like cluster	NRPS	Nidulanin A (50% of ge	ATEG_00228	ATEG_00220-ATEG_00	ATEG_00220-ATEG_002	ATEG_00227-ATEG_002	This study	
ATEG_00282 cluster	PKS	Pyranonigrin E (100% o	ATEG_00282	ATEG_00282-ATEG_00	ATEG_00276-ATEG_002	ATEG_00282	This study	
isoflavipucine cluster	Hybrid (PKS-NRPS)	Isoflavipucine biosynth	ATEG_00325	ATEG_00321-ATEG_00	ATEG_00316-ATEG_003	**ATEG_00325-ATEG_003**	Guo and Wang (2014) Front Microbiol 5.	
ATEG_00512 cluster	Terpene		ATEG_00512		ATEG_00507-ATEG_005	ATEG_00512	This study	
asterriquinones cluster	NRPS-like, DMAT	Terrequi biosynthetic g	ATEG_00700/ATEG_	ATEG_00696-ATEG_00	ATEG_00693-ATEG_007	ATEG_00700-ATEG_007	This study; Guo et al (2015) Chem sci 6, 5913-5921.	
ATEG_00821 cluster	ABBA-PT		ATEG_00821		ATEG_00815-ATEG_008	ATEG_00821	This study	
ATEG_00847/ATEG_00848 cluster	Betalactone		ATEG_00847/ATEG_00848		ATEG_00844-ATEG_008	ATEG_00847-ATEG_008	This study	
ATEG_00881 cluster	NRPS		ATEG_00881	ATEG_00881-ATEG_00	ATEG_00873-ATEG_008	ATEG_00881-ATEG_008	This study	
ATEG_00913 cluster	PKS	Pyranonigrin E (100% o	ATEG_00913	ATEG_00913	ATEG_00911-ATEG_009	ATEG_00913	This study	
ATEG_01002 cluster	NRPS		ATEG_01002	ATEG_01002-ATEG_01	ATEG_00996-ATEG_010	ATEG_01002-ATEG_010	This study	
ATEG_01052 cluster	NRPS-like		ATEG_01052	ATEG_01050-ATEG_01	ATEG_01044-ATEG_010	ATEG_01050-ATEG_010	This study	
abp cluster	DMAT		ATEG_01730	ATEG_01723-ATEG_01	ATEG_01725-ATEG_017	ATEG_01730	Guo et al (2015) Chem sci 6, 5913-5921.	
ATEG_01769 cluster	Terpene		ATEG_01769	ATEG_01769	ATEG_01767-ATEG_017	ATEG_01769	This study	
ATEG_01894 cluster	PKS		ATEG_01894	ATEG_01894	ATEG_01890-ATEG_019	ATEG_01894	This study	
aspulvinone E (hyphae) cluster	NRPS-like		ATEG_02004	ATEG_02004	ATEG_02001-ATEG_020	ATEG_02004	Guo et al (2015) Chem sci 6, 5913-5921.	
ATEG_02256 cluster	RiPP		ATEG_02256			ATEG_02256-ATEG_022	Nagano et al (2016) Fungal Genet Biol 86, 58-70.	
ATEG_02403 cluster	NRPS-like		ATEG_02403	ATEG_02403-ATEG_02	ATEG_02395-ATEG_024	ATEG_02403-ATEG_024	This study	
ATEG_02434 cluster	PKS		ATEG_02434	ATEG_02428-ATEG_02	ATEG_02426-ATEG_024	ATEG_02434	This study	
ATEG_02795 cluster	PKS		ATEG_02795		ATEG_02779-ATEG_028	ATEG_02792-ATEG_027	This study	
butyrolactones cluster	NRPS-like		ATEG_02815	ATEG_02815-ATEG_02	ATEG_02810-ATEG_028	ATEG_02815	Guo et al (2015) Chem sci 6, 5913-5921.	
ATEG_02831 cluster	NRPS, DMAT		ATEG_02823/ATEG_	ATEG_02815-ATEG_02	ATEG_02810-ATEG_028	ATEG_02818-ATEG_028	This study	
ATEG_02944 cluster	NRPS		ATEG_02944	ATEG_02944-ATEG_02	ATEG_02937-ATEG_029	ATEG_02944-ATEG_029	This study	
ATEG_03090 cluster	NRPS-like, DMAT		ATEG_03090/ATEG_	ATEG_03090-ATEG_03	ATEG_03082-ATEG_030	ATEG_03089-ATEG_030	This study	
ATEG_03255 cluster	RiPP		ATEG_03255			ATEG_03255	Nagano et al (2016) Fungal Genet Biol 86, 58-70.	
ATEG_03309 cluster	Terpene		ATEG_03309		ATEG_03305-ATEG_033	ATEG_03309	This study	
azaphilones cluster 1	PKS	Azaphilone (20% of ger	ATEG_03432/ATEG_	ATEG_03426-ATEG_03	ATEG_03423-ATEG_034	ATEG_03432-ATEG_034	Yin et al (2016) Appl microbiol biotechnol 100, 7787-7798.	
acetylaranotin cluster	NRPS	Acetylaranotin biosynt	ATEG_03470	ATEG_03462-ATEG_03	ATEG_03463-ATEG_034	**ATEG_03466-ATEG_034**	Guo and Wang (2014) Front Microbiol 5.	
ATEG_03528 cluster	NRPS		ATEG_03528	ATEG_03526-ATEG_03	ATEG_03520-ATEG_035	ATEG_03526-ATEG_035	This study	
aspulvinones (conidia) cluster	NRPS-like	Valactamide A (25% of	ATEG_03563	ATEG_03563	ATEG_03554-ATEG_035	ATEG_03563-ATEG_035	Guo et al (2015) Chem sci 6, 5913-5921.	
ATEG_03568 (facms0005) cluster	Terpene		ATEG_03568		ATEG_03554-ATEG_035	ATEG_03567-ATEG_035	Clevenger et al (2017) Nat chem biol 13, 895.	
valactamide A cluster	PKS, NRPS	Valactamide A (18% of	ATEG_03575/ATEG_	ATEG_03575-ATEG_03	ATEG_03572-ATEG_035	**ATEG_03574-ATEG_035**	Clevenger et al (2017) Nat chem biol 13, 895.	
pkb like cluster	PKS, NRPS-like		ATEG_03629/ATEG_	ATEG_03626-ATEG_03	ATEG_03623-ATEG_036	ATEG_03629-ATEG_036	Yin et al (2016) Appl microbiol biotechnol 100, 7787-7798.	
ATEG_04218 cluster	DMAT		ATEG_04218	ATEG_04218	ATEG_04215-ATEG_042	ATEG_04218	Wunsch et al (2015) Appl microbiol biotechnol 99, 1719-1730.	

ATEG_04322/ATEG_04323 cluster	NRPS, RiPP		ATEG_04322/ATEG_	ATEG_04315-ATEG_04	ATEG_04314-ATEG_043	ATEG_04321-ATEG_0432	This study; Nagano et al (2016) Fungal Genet Biol 86, 58-70.
aspterric acid cluster	Terpene	Aspterric acid (100% of	ATEG_04416		ATEG_04414-ATEG_044	ATEG_04415-ATEG_0441	Yan et al (2018) Nature 559, 415.
dehydrocurvularin cluster	PKS	Dehydrocurvularin bios	not annotated		ATEG_04552-ATEG_045	**ATEG_04563-ATEG_045**	Xu et al (2013) Appl Environ Microbiol 79, 2038-2047.
ATEG_04621 cluster	RiPP		ATEG_04621			ATEG_04621	Nagano et al (2016) Fungal Genet Biol 86, 58-70.
ATEG_04718 cluster	PKS		ATEG_04718	ATEG_04718-ATEG_04	ATEG_04713-ATEG_047	ATEG_04717-ATEG_0472	This study
ATEG_04748 cluster	RiPP		ATEG_04748			ATEG_04748	Nagano et al (2016) Fungal Genet Biol 86, 58-70.
ATEG_04975 cluster	NRPS-like		ATEG_04975	ATEG_04965-ATEG_04	ATEG_04966-ATEG_049	ATEG_04973-ATEG_0497	This study
ATEG_04999 cluster	DMAT		ATEG_04999	ATEG_04993-ATEG_04	ATEG_04997-ATEG_050	ATEG_04999-ATEG_0500	This study
ATEG_05073 (sidC like) cluster	NRPS		ATEG_05073/ATEG_	ATEG_05068-ATEG_05	ATEG_05066-ATEG_050	ATEG_05073-ATEG_0507	This study
ATEG_05795 cluster	NRPS-like		ATEG_05795	ATEG_05792-ATEG_05	ATEG_05788-ATEG_057	ATEG_05795	This study
ATEG_06056 cluster	PKS		ATEG_06056	ATEG_06056-ATEG_06	ATEG_06048-ATEG_060	ATEG_06056-ATEG_0605	This study
ATEG_06113 cluster	NRPS, DMAT		ATEG_06113/ATEG_	ATEG_06111-ATEG_06	ATEG_06107-ATEG_061	ATEG_06111-ATEG_0611	This study
DHN-type of pigment cluster	PKS	Naphthopyrone (100%	ATEG_06206	ATEG_06203-ATEG_06	ATEG_06197-ATEG_062	**ATEG_06203-ATEG_062**	Palonen et al (2017) Microorganisms 5, 22.
terreic acid cluster	PKS	Terreic acid biosynthet	ATEG_06275	ATEG_06272-ATEG_06	ATEG_06268-ATEG_062	**ATEG_06272-ATEG_062**	Guo and Wang (2014) Front Microbiol 5.
ATEG_06400 cluster	Terpene		ATEG_06400		ATEG_06397-ATEG_064	ATEG_06397-ATEG_0640	This study
ATEG_06680 cluster	PKS		ATEG_06680	ATEG_06678-ATEG_06	ATEG_06671-ATEG_066	ATEG_06678-ATEG_0668	This study
ATEG_06765 (mirC like) cluster	PKS		ATEG_06765	ATEG_06762-ATEG_06	ATEG_06761-ATEG_067	ATEG_06762-ATEG_0676	This study
ATEG_06998 cluster	NRPS-like		ATEG_06998	ATEG_06995-ATEG_06	ATEG_06991-ATEG_070	ATEG_06996-ATEG_0699	This study
ATEG_07026 cluster	RiPP		ATEG_07026			ATEG_07026	Nagano et al (2016) Fungal Genet Biol 86, 58-70.
ATEG_07067 (facms0007) cluster	PKS		ATEG_07067	ATEG_07062-ATEG_07	ATEG_07058-ATEG_070	ATEG_07062-ATEG_0706	Clevenger et al (2017) Nat chem biol 13, 895.
ATEG_07279/ATEG_07282 cluster	PKS		ATEG_07279/ATEG_	ATEG_07278-ATEG_07	ATEG_07271-ATEG_072	ATEG_07278-ATEG_0728	This study
ATEG_07310 cluster	RiPP		ATEG_07310			ATEG_07310	Nagano et al (2016) Fungal Genet Biol 86, 58-70.
ATEG_07358 (facms0016) cluster	NRPS		ATEG_07358	ATEG_07354-ATEG_07	ATEG_07351-ATEG_073	ATEG_07354-ATEG_0735	Clevenger et al (2017) Nat chem biol 13, 895.
ATEG_07367 cluster	Terpene		ATEG_07367		ATEG_07351-ATEG_073	ATEG_07362-ATEG_0736	This study
ATEG_07379 (facms0008) cluster	PKS, NRPS-like		ATEG_07379/ATEG_	ATEG_07374-ATEG_07	ATEG_07351-ATEG_073	ATEG_07374-ATEG_0738	Clevenger et al (2017) Nat chem biol 13, 895.
ATEG_07488 (sidD like) cluster	NRPS	Naphthopyrone (100%	ATEG_07488	ATEG_07485-ATEG_07	ATEG_07479-ATEG_075	ATEG_07488	This study
ATEG_07500 (facms0006) cluster	PKS	Duclauxin (14% of gene	ATEG_07500	ATEG_07500-ATEG_07	ATEG_07479-ATEG_075	ATEG_07500-ATEG_0750	Clevenger et al (2017) Nat chem biol 13, 895.
ATEG_07506 cluster	RiPP		ATEG_07505/ATEG_07506/ATEG_07507		ATEG_07479-ATEG_075	ATEG_07503-ATEG_0750	This study; Nagano et al (2016) Fungal Genet Biol 86, 58-70.
asperfuranone cluster	PKS	Asperfura biosynthetic	ATEG_07659/ATEG_	ATEG_07652-ATEG_07	ATEG_07652-ATEG_076	**ATEG_07659-ATEG_076**	Guo and Wang (2014) Front Microbiol 5.
ATEG_07806 cluster	RiPP		ATEG_07806			ATEG_07806-ATEG_0780	Nagano et al (2016) Fungal Genet Biol 86, 58-70.
ATEG_07894 cluster	NRPS-like		ATEG_07894	ATEG_07890-ATEG_07	ATEG_07887-ATEG_079	ATEG_07890-ATEG_0790	This study
ATEG_08172 cluster	PKS		ATEG_08172	ATEG_08167-ATEG_08	ATEG_08165-ATEG_081	ATEG_08167-ATEG_0817	This study
ATEG_08204 cluster	Terpene		ATEG_08204	ATEG_08204	ATEG_08200-ATEG_082	ATEG_08204	This study
ATEG_08348 cluster	NRPS-like		ATEG_08348	ATEG_08345-ATEG_08	ATEG_08341-ATEG_083	ATEG_08345-ATEG_0834	This study
astechrome cluster	NRPS, DMAT	Hexadehydro-astechro	ATEG_08427/ATEG_	ATEG_08424-ATEG_08	ATEG_08419-ATEG_084	**ATEG_08424-ATEG_084**	Bok et al (2015) BMC Genom 16, 343.
ATEG_08448 cluster	NRPS		ATEG_08448	ATEG_08438-ATEG_08	ATEG_08439-ATEG_084	ATEG_08443-ATEG_0844	This study
geodin cluster	PKS	Trypacidin biosynthetic	ATEG_08451	ATEG_08438-ATEG_08	ATEG_08439-ATEG_084	**ATEG_08449-ATEG_084**	Nielsen et al (2013) PloS one 8.
azaphilones cluster 2 pki like	PKS	Azaphilone biosyntheti	ATEG_08662	ATEG_08653-ATEG_08	ATEG_08654-ATEG_086	ATEG_08662-ATEG_0866	Yin et al (2016) Appl microbiol biotechnol 100, 7787-7798.
ATEG_08678 cluster	NRPS-like		ATEG_08678	ATEG_08678	ATEG_08669-ATEG_086	ATEG_08678	This study
ATEG_08706 cluster	RiPP		ATEG_08706			ATEG_08706-ATEG_0870	Nagano et al (2016) Fungal Genet Biol 86, 58-70.
ATEG_08827 cluster	RiPP		ATEG_08827			ATEG_08826-ATEG_0882	Nagano et al (2016) Fungal Genet Biol 86, 58-70.
phenguignardic acid cluster	NRPS-like		ATEG_08899	ATEG_08899	ATEG_08895-ATEG_089	**ATEG_08899***	Sung et al (2017) Fungal genet biol 101, 1-6.
ATEG_08904 cluster	Terpene		ATEG_08904	-	ATEG_08895-ATEG_089	ATEG_08904	This study
ATEG_09019 cluster	NRPS	Phomopsins (100% of g	ATEG_09019	ATEG_09007-ATEG_09	ATEG_09010-ATEG_090	ATEG_09010-ATEG_0901	This study
ATEG_09033 cluster	NRPS-like		ATEG_09033	ATEG_09028-ATEG_09	ATEG_09010-ATEG_090	ATEG_09028-ATEG_0903	This study
asperphenamate cluster	NRPS	Asperphenamate (100%	ATEG_09064/ATEG_	ATEG_09059-ATEG_09	ATEG_09056-ATEG_090	ATEG_09061-ATEG_0907	Li et al (2018) Chem sci 9, 2589-2594.
ATEG_09088 cluster	PKS		ATEG_09088	ATEG_09082-ATEG_09	ATEG_09078-ATEG_091	ATEG_09088-ATEG_0909	This study
ATEG_09100 cluster	PKS		ATEG_09100	ATEG_09082-ATEG_09	ATEG_09078-ATEG_091	ATEG_09100-ATEG_0910	This study
ATEG_09142 cluster	NRPS-like	Notoamide (11% of ger	ATEG_09142	ATEG_09142-ATEG_09	ATEG_09135-ATEG_091	ATEG_09142-ATEG_0914	This study
ATEG_09295 cluster	Siderophore		ATEG_09295	-	ATEG_09294-ATEG_092	ATEG_09294-ATEG_0929	This study
citreoviridin cluster	PKS	Citreoviridin biosynthet	ATEG_09617	ATEG_09611-ATEG_09	ATEG_09610-ATEG_096	**ATEG_09616-ATEG_096**	Lin et al (2016) Org lett 18, 1366-1369.
lovastatin cluster	PKS	Lovastatin biosynthetic	ATEG_09961/ATEG_	ATEG_09960-ATEG_09	ATEG_09958-ATEG_099	**ATEG_09960-ATEG_099**	Guo and Wang (2014) Front Microbiol 5.
ATEG_09980 cluster	DMAT		ATEG_09980	ATEG_09980-ATEG_09	ATEG_09978-ATEG_099	ATEG_09980	Wunsch et al (2015) Appl microbiol biotechnol 99, 1719-1730.
ATEG_10075 cluster	Terpene	Deoxysambucinol / san	ATEG_10075	-	ATEG_10071-ATEG_100	ATEG_10075	This study
terretonin cluster	PKS	Terretonin biosynthetic	ATEG_10080	ATEG_10079-ATEG_10	ATEG_10071-ATEG_100	**ATEG_10077-ATEG_100**	Guo and Wang (2014) Front Microbiol 5.
ATEG_10296/ATEG_10297 cluster	Betalactone		ATEG_10296/ATEG_	-	ATEG_10293-ATEG_103	ATEG_10296-ATEG_1029	This study
epi-aszonalenins cluster	NRPS, DMAT	Acetylaszonalenin bios	ATEG_10305/ATEG_	-	ATEG_10293-ATEG_103	ATEG_10304-ATEG_1030	This study
